# Recent advances in regenerative biomaterials

**DOI:** 10.1093/rb/rbac098

**Published:** 2022-12-05

**Authors:** Dinglingge Cao, Jiandong Ding

**Affiliations:** State Key Laboratory of Molecular Engineering of Polymers, Department of Macromolecular Science, Fudan University, Shanghai 200438, China; State Key Laboratory of Molecular Engineering of Polymers, Department of Macromolecular Science, Fudan University, Shanghai 200438, China

**Keywords:** regenerative biomaterial, tissue induction biomaterial, medical material, tissue regeneration

## Abstract

Nowadays, biomaterials have evolved from the inert supports or functional substitutes to the bioactive materials able to trigger or promote the regenerative potential of tissues. The interdisciplinary progress has broadened the definition of ‘biomaterials’, and a typical new insight is the concept of tissue induction biomaterials. The term ‘regenerative biomaterials’ and thus the contents of this article are relevant to yet beyond tissue induction biomaterials. This review summarizes the recent progress of medical materials including metals, ceramics, hydrogels, other polymers and bio-derived materials. As the application aspects are concerned, this article introduces regenerative biomaterials for bone and cartilage regeneration, cardiovascular repair, 3D bioprinting, wound healing and medical cosmetology. Cell-biomaterial interactions are highlighted. Since the global pandemic of coronavirus disease 2019, the review particularly mentions biomaterials for public health emergency. In the last section, perspectives are suggested: (i) creation of new materials is the source of innovation; (ii) modification of existing materials is an effective strategy for performance improvement; (iii) biomaterial degradation and tissue regeneration are required to be harmonious with each other; (iv) host responses can significantly influence the clinical outcomes; (v) the long-term outcomes should be paid more attention to; (vi) the noninvasive approaches for monitoring *in vivo* dynamic evolution are required to be developed; (vii) public health emergencies call for more research and development of biomaterials; and (viii) clinical translation needs to be pushed forward in a full-chain way. In the future, more new insights are expected to be shed into the brilliant field—regenerative biomaterials.

## Graphical Abstract

**Figure:**
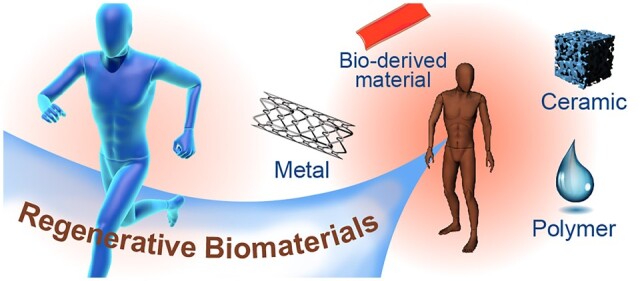

## Introduction

Biomaterials have been developed from simply implanting to tissue engineering and regenerative medicine [1, 2]. Some tissues in human body have the ability of regeneration after suffering damage. However, most tissues are unable to spontaneously achieve complete healing in many cases like severe traumatic injury, degenerative disease or infection, which thus requires clinical intervention. Before the concept of regenerative biomaterials, the early generation of biomaterials is bioinert and simply regarded as a structure support or a medicine repository. At this stage, the regeneration process relies, if any, on the innate potential of body instead of biomaterials. With the development of biomaterials and biology, researchers start to add active substances including drugs and living cells in materials [3, 4]. The cell-laden biomaterials should, in the classic tissue engineering, be constructed *in vitro* and then implanted into body, which is difficult due to the complex cell culture procedure and low engraftment efficacy. With the in-depth understanding of medicine and biomaterials, it is realized that the regeneration process of a damaged cell or a tissue is complex and the interactions between cells and surroundings are crucial for regeneration [5, 6]. Modern regenerative biomaterials are inspired from that some materials can regulate molecular signal pathway and cellular behavior, indicating their potential capacity of directing the process of tissue regeneration without active drugs and cells [6]. Increased research interest is devoted to the new generation of regenerative biomaterials [7–10]. The regenerative biomaterials serve as not only a material for a structure support or a delivery vehicle but also a functional regulator [10–13].

From the narrow sense, the term ‘regenerative biomaterials’ refers to the materials that have the ability to regenerate the damaged tissues and organs. In particular, the concept of tissue induction biomaterials has shed new insight into the field of biomaterials [14–19]. According to the latest consensus of definition of biomaterials and relevant key terms by The International Union of Societies for Biomaterials Science and Engineering (IUSBSE), a tissue induction biomaterial is ‘a biomaterial designed to induce the regeneration of damaged or missing tissues or organs without the addition of cells and/or bioactive factors’, while a biomaterial is defined as ‘a material designed to take a form that can direct, through interactions with living systems, the course of any therapeutic or diagnostic procedure’ [20]. So, the term ‘regenerative biomaterials’ is close to tissue inducing biomaterials in the narrow sense, and also to the new definition of biomaterials in the broad sense.

This review employs its broad sense. With the development of advanced regenerative biomaterials, researchers have recognized that only the interdisciplinary coordination could unlock the full potential of regenerative biomaterials. The term regenerative biomaterials and thus the topics of this review are not limited to tissue induction biomaterials. We will summarize the progress of medical materials ranging from the facilitated biomaterials to repair, replace or regenerate tissues, to the advanced biomaterials to improve human life quality.

**Figure 1.**
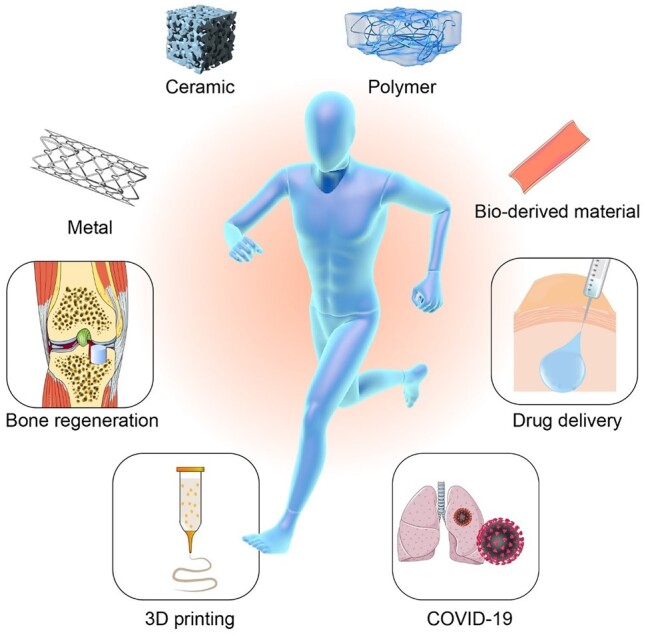
Different sources and applications of regenerative biomaterials.

Different sources and applications of regenerative biomaterials are schematically presented in Fig. 1. In this article, we attempt to provide a comprehensive and multidisciplinary insight into regenerative biomaterials, and aim to provide a broad overview of the recent achievements in this exciting field for broader audiences. First, we introduce the advances of different sources of biomaterials in recent years, compare the advantages and shortcomings of these materials, and describe the advanced design using these materials. Second, we highlight the recent progress on emerging applications of regenerative biomaterials in different tissues. Third, we review the studies on the interactions of biomaterials with cells and tissues. In particular, we briefly introduce the biomaterials related to the application in public health emergency, especially the biomaterials associated to coronavirus disease 2019 (COVID-19). Finally, we point out the challenges which should be paid more attentions for research and development (R & D) of regenerative biomaterials and pertinent fields in the future.

## The different sources of biomaterials for tissue regeneration

Materials are classified into metals, nonmetallic inorganics and polymers. Considering the characteristics of medical materials, we describe the regenerative biomaterials in sequence of metals, nonmetallic inorganics, hydrogels, other polymers and bio-derived materials. Here, polymers are divided into two parts owing to their abundant types and rich performances, and bio-derived materials could be regarded as a special composite medical material. Each of them has the distinctive advantages and limitations.

### Metals

Metallic biomaterials have been widely used clinically owing to the outstanding mechanical property and excellent durability for reconstruction and regeneration. Taking titanium (Ti) as an example, it is a useful material for orthopedic implants with excellent mechanical properties and high corrosion stability. Recent studies focus on surface modification, alloying or other approaches to enhance the material performance [21–25]. For instance, roughening of surfaces is thought to promote osteointegration by increasing the apposition of osseous tissue and favoring epithelial attachment to the implant [26]. Surface micro/nano structures can be endowed on titanium via grit-blasting and acid-etching, laser method and electrochemical method [27–29]. In addition to change the texture of Ti implants, surface modification using bioactive components can also improve the interaction between materials and cells [30]. For example, Sun and Wang [31] groups used extracellular vesicle coating to endow a Ti implant with biological activities. Ti exhibits poor wear resistance and is not suitable for articulating surfaces. But the thick film composed of titanium oxides on the surface could separate Ti implants from surroundings and realize high corrosion resistance. Some composite materials have been designed to overcome the shortcomings of a single-component material. Introducing some alloying elements in Ti materials to fabricate a binary Ti alloy is helpful for improving a titanium-based material. For instance, adding niobium (Nb) into a Ti material can improve the tensile performance [32].

**Figure 2.**
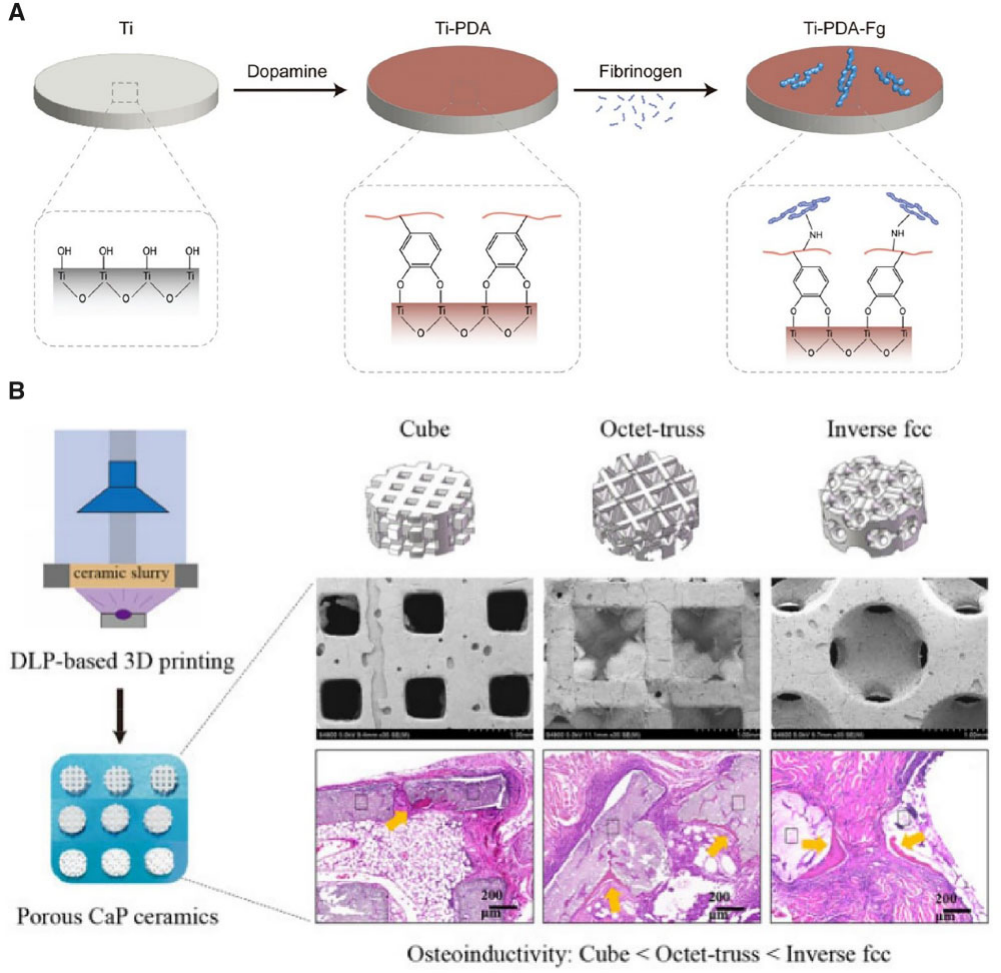
Metal and ceramic implants. (**A**) A fibrinogen-modified titanium alloy as a model material for an orthopedic percutaneous medical device. Reproduced from Ref. [36] with permission of Royal Society of Chemistry, © 2022. (**B**) Porous CaP ceramic scaffolds with distinct structures fabricated via digital light processing (DLP)-based 3D printing. Reproduced from Ref. [37] with permission of Oxford Press, © 2022.

The most widely used titanium-relevant medical alloy is Ti6Al4V, which is regarded as a good alternative material for percutaneous or transcutaneous devices owing to its high mechanical strength and excellent corrosion resistance. Like Ti implants, Ti6Al4V also needs surface modification to improve cell–material interactions for osseointegration and anti-inflammatory [33, 34]. In particular, the biological sealing can be improved via surface modification [35, 36]. Unlike a porous scaffold [37], titanium and its alloys are applied as a bulk material as usual, yet still with a large amount of surfaces exposed to the tissue environment and faced with a challenge of biological sealing. Ding *et al*. [36] fabricated a fibrinogen-modified Ti6Al4V implant and resulted in better biological sealing and osseointegration. Considering that proteins are hard to be bound in a metal, they employed a polydopamine (PDA) coating to mediate the covalently binding between the protein and the metal, as schematically indicated in Fig. 2A. Since the pioneering work of Messersmith group [38], mussel-inspired surface chemistry has been extended in multifunctional coatings owing to the amino and catechol groups and abilities for self-polymerization and spontaneous deposition [38–42]. Surface modification of metals is able to influence various cell behaviors on materials.

The overstable materials like Ti do not match the dynamic process of tissue regeneration in many cases, and may need to be removed sometimes by a secondary surgery, which increases the pain and cost of patients [43]. Therefore, biodegradable metals hold promises for tissue regeneration. A part of metals has the corrosion and bioresorption characteristics *in vivo*, enabling them as degradable biomaterials. Nevertheless, Ma *et al*. [44] reviewed numerous publications and found that the outcomes of some degradable metals for bone repairing in animal models might be overestimated, as evidenced by that the early fracture repair performance of degradable metals was worse than that of non-degradable metals owing to the weaker fixation ability. The degradation time differs with metal types. For example, magnesium (Mg) materials degrade a few months, while raw iron (Fe) materials degrade over a few years. Bone tissue healing usually require more than 12 weeks, and thus the degradation of Mg is too quickly to match the process of new bone formation. In other words, the fast degradation may impede the bone fracture healing when applied in the bone regeneration. Similarly, the biodegradable performance could be hindered by the overly slow corrosion of metal biomaterials when used for cardiovascular treatments. Therefore, tuning the degradation rate of metal to a proper time range is important for biomedical applications. Researchers have come up with some strategies to achieve the expected degradation rate of metallic materials. Alloying is a conventional method to adjust the properties of metallic biomaterials. For example, Wang *et al*. [45] used magnesium–strontium alloy to optimize the corrosion resistance of the material in a canine mandibular defect model. As reviewed by Li and Ma groups [44], it is still hard for an alloy to exhibit *in vivo* degradation with its rate exactly matching the regeneration process of bone tissue.

It is a frontier to develop more strategies to adjust corrosion rates of metals under biomimetic condition. In this aspect, one of typical progress in recent years comes from the concept of metal–polymer composite stent (MPS) [46]. It is interesting that a polylactide (PLA) coating on iron did not protect Fe from its corrosion, but accelerated Fe corrosion owing to the interference of Ca-P precipitating on Fe and the local acidic microenvironment on the Fe surface along with hydrolysis of the aliphatic polyester, as revealed by Ding’s group [47]. More strategies to regulate corrosion of biometals have been suggested [48, 49], and still in progress. For example, Yuan *et al*. [50] applied the Ca-P coating to overcome the rapid degradation of Mg-based implants, and the clinical study confirmed the effectiveness and safety. Similarly, micro-arc oxidation was used to produce a ceramic oxide layer on Mg-based implants to improve the corrosion resistance [51].

With the material degradation, the metallic ions were released from the metals. Many metals contain the important elements required by human body including Fe, calcium (Ca), copper (Cu) [52] and Mg [53]. Therefore, taking advantage of the released ions could endow biomaterials more performances [54]. For example, Mg has the potential of osteogenic and angiogenic properties [55]. Mg could facilitate bone regeneration by proliferating osteoblasts, promoting osteogenic differentiation and achieving mineralization.

While the released ions from metallic biomaterials might be helpful for human body to biological functions, the excessive release of metallic ions is harmful. For example, Mg-related biomaterials can cause side effects such as cytotoxicity [56]. In addition, it was found that Mg alloy could, by significantly affecting adhesion and migration of endothelial cells (ECs), inhibit the process of re-endothelialization of Mg stents [57]. Owing to superelasticity, nickel-titanium (nitinol, NiTi) has been widely used in biomedicine, particularly as medical devices for interventional treatment. However, the US Food and Drug Administration (FDA) has warned the risk of the released nickel ions. After years of efforts and cooperation among university, hospital and company, a nanocoating using TiN was found to significantly alleviate the content of released Ni^+^, and the corresponding advanced occluder based on a Chinese core material technique has recently been commercialized in many countries [58].

Poor cytocompatibility is not always a disadvantage [59]. The cytotoxicity of the dissolution of bioactive ions can also serve significant antibacterial activities like silver (Ag), Cu and Mg [60–63], which is beneficial to tissue regeneration owing to the anti-infection capability. Bolzoni *et al*. [64] added Cu to titanium alloys via powder metallurgy to prevent bacterial infection during surgical implantation. Zhao *et al*. [65] used Ag–selenium (Ag–Se) nanocomposite coating to inhibit bacterial adhesion of implants.

In addition to the antibacterial function, metal ions can also be employed in anti-inflammation. For example, Gao *et al*. prepared nanosized zinc-based metal–organic frameworks (MOFs) with favorable antibacterial and anti-inflammatory properties via slowly releasing Zn ions [66]. Some metallic materials can be utilized in tumor ablation for their superior photothermal conversion efficiency [67]. Moreover, some rare-earth metals gradually raised researchers’ attentions as biomaterials owing to their unique electronic configurations and variable valence states. For example, ceria was utilized in industrial catalysis and has recently been developed in the form nanoceria to scavenge reactive oxygen and nitrogen species [68]. Cerium can flip-flop between valence states of Ce^3+^ (reduced) and Ce^4+^ (oxidized), which enables ceria to form oxygen vacancies in the lattice structure. Nanoceria is able to form more oxygen vacancies because the decreased surface area to volume ratio. Besides, some rare-earth metal-based materials have the magnetic properties, which can be used in magnetic resonance imaging (MRI), such as gadolinium [69], holmium and dysprosium [70].

Moreover, some metal substances are able to catalyze proteins to participate the life process. For example, copper has the potential to catalyze nitric oxide (NO) donors to release NO. Wang *et al*. [71] designed a copper-loaded PDA coating for implanted blood contact materials to achieve an antithrombotic function via sustained release of NO. Hence, with the development of regenerative medicine, metals have never simply been a mechanical support but also a functional source.

### Nonmetallic inorganics

Nonmetallic inorganics contains two classes, crystalline ceramics and amorphous glass. They are particularly useful for orthopedic tissue regeneration. Ceramics are divided into bioinert ceramics and bioactive ceramics. Bioactive ceramics can induce body to generate biological responses after implantation [72, 73]. Alumina and zirconia are the bioinert ceramics. Calcium phosphate ceramics, especially tricalcium phosphate, biphasic calcium phosphate and hydroxyapatite, have been widely used in bone regeneration owing to biomimetic chemical composition and their osteoinductive and biodegradable abilities [74]. The osteoinductive activity of calcium phosphate ceramics was first explicitly recognized by Zhang [75] and Ripamonti [76] in 1991. Afterwards, some inorganic scaffolds were found to facilitate new bone deposition on the surface [77]. The underlying mechanisms of stimulating bone regeneration with bioactive ceramics have also been investigated [78–81].

Bioactive ceramics not only have functions to directly simulate osteogenic differentiation of mesenchymal stem cells (MSCs) but also have, sometimes, immunomodulatory ability for creating a favorable inflammation microenvironment for bone regeneration. After the facture, inflammatory macrophage (M1 phenotype) is able to amplify the inflammatory cascade, which plays an important role in bone healing in the initial stage. The inflammatory microenvironment changes during bone healing. Therefore, the M1 macrophage is required to be timely regulated and converted to the M2 phenotype for better bone regeneration. Chang *et al*. [82] found that ionic products of calcium silicate could enhance the immunosuppressive function of human bone marrow-derived MSCs. Zhang *et al*. [83] found that dicalcium silicate could promote osteogenic differentiation of mouse bone marrow-derived MSCs via inducing macrophagic inflammation (M1 phenotype). Specifically, the release calcium and silica ions from dicalcium silicate could affect mitochondrial function and induce autophagy in RAW264.7 cells, leading to M1 polarization.

Based on the fundamental studies of mechanisms, the *in vivo* efficiency of ceramics has also been confirmed. Recently, calcium phosphate ceramics has achieved regenerative bone repair for large segmental bone defects in a goat model [84]. A porous structure is important for the biological activities of a scaffold, because the pores in the scaffold could favor the growth of the surrounding cells and the dissolution of ionic compounds. The pore size, porosity and interconnectivity of a porous scaffold influence the efficacy of bone regeneration. Briefly, the pore size should allow for the migration, proliferation and differentiation of cells in the scaffold [85]. An increased porosity decreases the mechanical performance of the scaffold, and an inadequate porosity leads to poor regeneration [86, 87]. The pore geometry might act as a mechanical cue to influence cell behaviors [88]. As shown in Fig. 2B, Zhu *et al*. [37] compared the osteoinductive effects of various CaP ceramic scaffolds with distinct structures of cube, octet-truss, inverse face-centered cube (fcc) and foam. They found that the scaffolds with a foamlike structure showed strongest osteoinduction owing to the local high ionic microenvironment caused by some non-through holes and smaller pore diameter. The inverse fcc group showed the higher osteoinductive ability with their spherical pore structure compared to cube and octet-truss.

The traditional ceramics still suffer from some drawbacks such as intrinsic brittleness. The processability of a ceramic is usually not as convenient as a polymer. So, one has tried to combine ceramic and polymer to make a composite, and an appropriate compositing not only improves the compressive strength of ceramics but also improves the toughness of the polymers [89]. For example, Duan *et al*. [90] coated hydroxyapatite with bone morphogenic protein 2-loaded poly(L-lactide) (PLLA) fibers via electrospinning without obstructing the pore interconnectivity. The drug-loaded polymeric coating of the scaffold improved the compressive strength and osteogenesis ability of the scaffold. Besides, Habibovic *et al*. [91] developed a ceramic sponge that consisted of a self-supporting network of seamlessly interwoven hydroxyapatite nanowires and tricalcium phosphate nanofibers; this method showed excellent processability into different shapes and dimensions.

Bioactive glasses are another important class of nonmetallic inorganic biomaterials and can bind to bone and elicit biological effects by releasing biologically active ions of some elements including silicon (Si), Ca, phosphate (P) [92]. The bioactive glass of the first generation is 45S5 (Bioglass^®^). It was invented by Larry Hench in 1969 and has been clinically used for bone repairing for many years [93]. Recently, the biomaterials based on bioactive glass have been optimized to exert various biological functions. More biologically active elements were induced in bioactive glass. For instance, strontium-incorporated bioactive glass was developed to improve the bone repair function via sustained release of strontium [94]. Chen *et al*. [95] fabricated Cu/Ca-impregnated bioactive glass nanoparticles via incorporating copper/calcium in the SiO_2_ frameworks for the treatment of osteosarcoma. The level of reactive oxygen species (ROS) in tumor cells could be synergistically enhanced via Fenton-like reaction induced by copper ions and the released calcium ions. At the same time, the formation of hydroxyapatites induced by bioactive glass and the released calcium ions could synergistically lead to calcification. Furthermore, the enhanced ROS level and calcification led to tumor cell death.

### Hydrogels

A hydrogel is a kind of polymeric materials conserving a large amount of water in a 3D network. Hydrogels exhibit unique properties and have been applied in the biomaterial field [96–100]. For example, a hydrogel stands out as a kind of soft materials owing to the moderate mechanical strength, which has the high compatibility with soft tissues [101, 102]. Therefore, hydrogels have been used as tissue fillers and tissue adhesives [103, 104]. The feature of high-water content makes hydrogels quite friendly to biological environments [105]. Some polymeric hydrogels are of excellent degradability and biocompatibility [106, 107]. Notably, the 3D network endows hydrogels with the capacity of transport, storage and controlled release of drugs [108–110]. Yu *et al*. [111] fabricated an injectable thermogel applied in breast-conserving surgery, which could not only act as a temporary filling material but also prevent the local relapse via sustained release of Herceptin. The most convenient approach of loading a drug into a hydrogel is directly mixing, and the release manner depends on multiple aspects [112–114]. Generally, macromolecular drugs such as proteins can be released in a sustained manner relatively easily owing to their similar sizes with the mesh of a hydrogel [115–117], while water-soluble small molecular drugs lead to a significant burst release as usual [118, 119]. Sometimes, the small molecular drugs can achieve a sustained release in a hydrogel via transformation of their condense-state physical forms such as crystallization [120, 121].

Except the cargo size, the hydrophilic-hydrophobic extent of a loaded drug decides the release manner. For example, gemcitabine is a potent anti-tumor drug, but the short half-life of this hydrophilic drug has severely limited the clinical application. Yu *et al*. [122] modified gemcitabine with fatty acids to increase its hydrophobicity, and subsequently loaded it into a hydrogel. The release of the modified gemcitabine out of the hydrogel was prolonged up to 37 days, while unmodified gemcitabine in the hydrogel exhibited a severe burst release and nearly 70% of that was released on the first day. The hydrophilic drug can also achieve a sustained release in appropriate hydrogels via interaction with the networks. For example, lixisenatide is a hydrophilic peptide, one of the family of glucagon-like peptide-1 receptor agonists, and has been licensed by FDA for the treatment of type 2 diabetes mellitus. Yu *et al*. [123] leveraged the negative charge of their synthetized biodegradable polymer and the positive charge of lixisenatide to achieve sustained release by simply blending the peptide with the aqueous polymer solutions.

The network in a hydrogel can be crosslinked by the chemical bonds or physical interactions, resulting in, respectively, a chemical hydrogel and a physical hydrogel [124, 125]. Some hydrogels could be formed by physical stimuli free of chemical reaction and thus particularly useful in biomedicine [126, 127]. As an example of chemical hydrogels, the photo-crosslinkable hyaluronic acid hydrogel was formed under an ultraviolet radiation, and porcine cartilage regeneration was achieved after combined with platelet-rich plasma in a porcine model [128].

**Figure 3.**
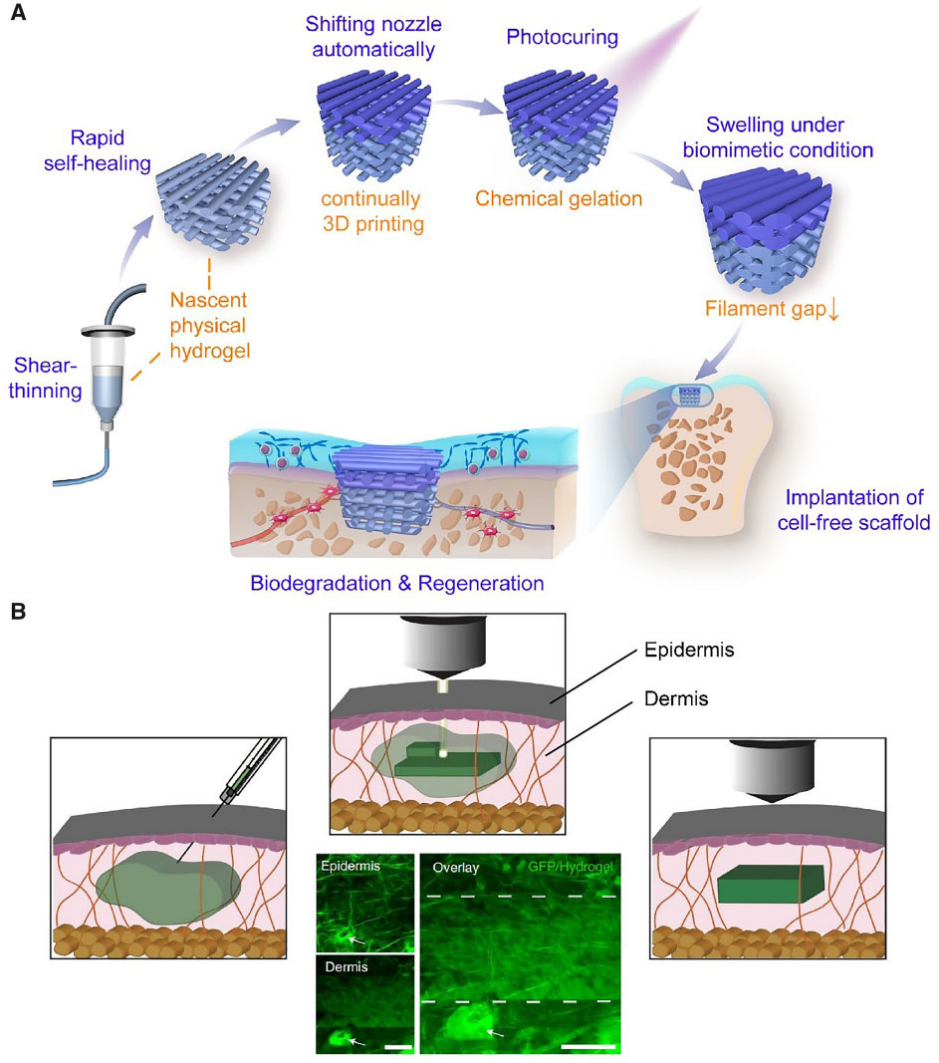
Hydrogels used in 3D printing. (**A**) Schematic diagrams of the key material properties to enable the continuous 3D-printing of the bilayered hydrogel scaffold. Reproduced from Ref. [129] with permission of Wiley-VCH, © 2020. (**B**) Schematic and photographs showing two-photon crosslinking of a hydrogel into dermis across the epidermis. Scale bars, 100 µm. Adapted from Ref. [133] with permission of Springer Nature, © 2022.

If a flowable aqueous solution rapidly gels upon a stimulus, the material system can be applied in 3D printing. Gelatin methacryloyl (gelMA) is a biomacromolecule-derived macromonomer, and its polymerization leads to a light-curable hydrogel denoted, a bit differently, as GelMA, as suggested by Ding *et al*. [129]. The macromonomer and its resultant hydrogel have been widely used in 3D printing [130–132]. Recently, Ding *et al*. [129] fabricated a continuous 3D printing of a bilayered scaffold combined with the sol-gel transition of the aqueous solution of gelMA and the photocrosslinking of the gelMA macromonomer. As shown in Fig. 3A, such a bilayered scaffold was printed by extruding a nascent physical hydrogel, taking advantage of non-Newtonian and thermoresponsive rheological properties of the aqueous solution of gelMA. The resultant hydrogel scaffold is promising as a biomaterial to regenerate articular cartilage.

The conventional 3D printing biomaterials were usually shaped by layer-by-layer positioning *in vitro*, and then implanted *in vivo* by surgery. However, in a recent study, Elvassore *et al*. [133] injected photo-active polymers to achieve an intravital 3D bioprinting by fabricating *in situ* 3D constructs in live animals without surgical procedures. After screened many coumarin derivatives, they chose 7-hydroxycoumarin-3-carboxylate–poly(ethylene glycol) conjugates as the raw material for their 3D bioprinting. As shown in Fig. 3B, they took advantage of multiphoton exciting to accurately form a photo-crosslinking hydrogel inside dermis, skeletal muscle and brain. The multiphoton microscopy used femtosecond pulsed infrared laser light (two-photon excited wavelength >850 nm) to enable sufficient penetration as well as high resolution. Compared to the conventional 3D printing, the intravital 3D bioprinting could achieve the printing process without surgical procedure and could realize real-time imaging. Gou *et al*. [134] also reported a technique of noninvasive *in vivo* 3D bioprinting. They subcutaneously injected the bioink composed of a monomer solution and stem cells and then fed the images of the scaffold to a computer. The digital near-infrared light was dynamically generated by the connected micromirror device chip and timely projected to noninvasively induce the spatial polymerization of the local injected bioink. After months, the prior injected stem cells were able to form a complex tissue or organ. Notably, they printed an ear-shaped construct using such a technique in BALB/c nude mice.

As a unique kind of injectable hydrogels, thermosensitive hydrogels have been extensively studied since Kim *et al*. [135] reported a novel block copolymer consisted of poly(ethylene oxide) (PEO) and PLLA in 1997. The biodegradable feature of this block copolymer distinguishes itself from other thermosensitive polymers, such as poly(N-isopropyl acrylamide) (PNIPAM) and poly(epoxy oxide)-*b*-poly(propylene oxide)-*b*-poly(epoxy oxide) (PEO-*b*-PPO-*b*-PEO). Several block copolymers based on polyether-polyester have been synthesized and exhibit the capability to be physically gelled upon heating, which can be a low-viscous sol at room temperature, and form a gel at body temperature (about 37°C) [136–139].

Ding group has systematically investigated the thermogels composed of the block copolymers of poly(ethylene glycol) (PEG) and poly(lactide-*co*-glycolide) (PLGA) [140–144]. The gelation mechanism of such amphiphilic copolymer system in water has been uncovered by Ding *et al*. [145]. The emergence of the sol-gel transition requires suitable molecular weight [146], the ratio of hydrophilic/hydrophobic blocks [147], sequences of the units of lactic acid and glycolic acid [148], side chains [149] and molecular weight distribution [150, 151], which results in a narrow gelation window for one-component copolymer system. To solve this problem, Ding group put forward a ‘block blend’ strategy using two copolymers with different hydrophilic/hydrophobic ratios to well tune the gelation window [152]. Both PEG and PLGA have been approved by FDA and have been applied clinically for many years, but until now, none of their block copolymers has been approved as a medical material in any country. Therefore, the clinical translation of this raw material in the future will be much valuable. Very recently, Ding’s group realize the dissolution of PLGA-PEG-PLGA block copolymers within half minute via manual shaking using a calcium coordination strategy [153]. While some additives could influence the performances of PLGA-PEG-PLGA-based hydrogels [154], appropriate small molecular soluble additives could accelerate the dissolution of chain-like polymers and meanwhile maintain the thermogellability.

### Other polymers

Polymer is the most giant family of biomaterials [155, 156]. In addition to hydrogels composed of cross-linked polymer networks, most of polymers exhibit excellent processability into various macroscopic physical forms. For example, poly(methyl methacrylate), named bone cement, has been used as a filling material to achieve bone repairing [157–159]; synthetic polyetheretherketone (PEEK) has been applied in orthopedics [160]; polytetrafluoroethylene has been used as an implantable material owing to its excellent biocompatibility and mechanical properties [161]. Besides the nonbiodegradable polymers as mentioned above, biodegradable polymers have been used in clinic [162]. For example, PLGA and PLLA, two of the most successfully developed biodegradable polymers, have been widely applied in drug delivery [163, 164] and cardiovascular stents [165].

Lack of biological recognition is one of the shortcomings of most of synthetic polymers. To address such issue, physical and chemical modifications have been developed to endow polymer materials with bioactivity [166, 167]. Immobilizing integrin’s ligands to promote biological recognition such as promoting cell adhesion, migration and differentiation, is a powerful approach of biomaterial modification [168]. Arginine-glycine-aspartic acid (RGD) peptides is the most popular ligand for improving cell adhesion, which can be immobilized into polymer molecules via chemical synthesis [169, 170]. Besides, in order to realize well-controlled surface modification of biomaterials, nanolithography and micropatterning techniques have been developed [171]. Taking advantage of photolithography techniques, gold nanoarrays on substrate material were obtained and subsequently grafted with RGD peptides [172]. Compared to polymeric molecular modification, such functionalized patterns can better control the spatial distribution on the microscale and nanoscale.

Some natural polymers are with good biocompatibility, biodegradability, weak immunogenic effects and cost-effectiveness [173, 174]. Typical examples are silk fibroin [175, 176], chitosan [177–179], hyaluronic acid [180, 181] and alginate [182]. Silk fibers were originally used as suture biomaterials owing to their unique mechanical properties [183]. With the insight into silk fiber construction, silk fibroin, the core component in silk fiber, is a kind of important natural protein polymer with great potentials in biomaterials [184]. In addition to the commercialized silk suture products, silk can be applied in drug delivery and tissue repairing for its controllable degradation behavior and good biocompatibility [185, 186]. Irvine *et al*. [187] used silk fibroins as tips to fabricate a microneedle patch with encapsulating stabilized human immunodeficiency virus (HIV) immunogen and adjuvant, and the degrees of β-sheet crystallinity of silk proteins can regulate the release rate. Alginate is a natural anionic polymer and has been proved as food additive and pharmaceutical excipient. Its aqueous solution can transform into a hydrogel in the presence of divalent or multivalent cations such as Ca^2+^ [188]. Alginate hydrogels have also been extensively investigated in the fields of drug delivery and tissue repairing. Moony group has developed an alginate cryogel with interconnected porosity via the cryogelation of methacrylated alginate [189]. The resultant microporous alginate cryogel differs with conventional alginate hydrogels in that it can be readily compressed and injected through a surgical needle for delivery owing to its elastic sponge-like property [190]. They used the cryogel to fabricate microporous-biomaterial vaccine via pre-loading tumor cells or cytokines to boost immune response [191, 192]. Besides, some biopolymers, such as polypeptides and nucleic acids have showed great potential in the biomaterial field [193].

Compared to other material classes especially metals, polymers are sometimes faced with insufficient mechanical properties [194]. In order to enhance the mechanics of polymers, one way is to develop new polymers; the other way is to optimize the condensed-state properties; the third way is the introduction of a polymer-based composite. Luo *et al*. [195] used laponite as the filler in a 3D-printed polycaprolactone scaffold to regulate the stiffness for bone regeneration. Liu *et al*. [196] fabricated a composite scaffold by embedding nanohydroxyapatite in a collagen hydrogel to improve the mechanical properties.

**Figure 4.**
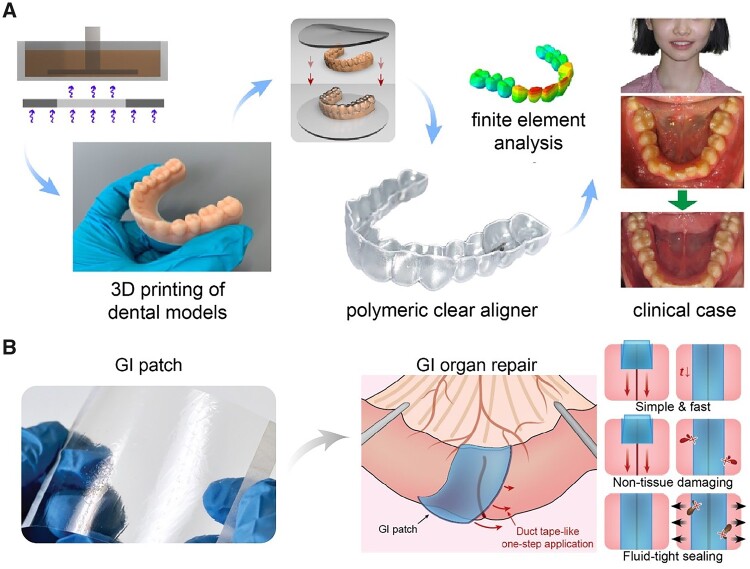
Some polymer-based biomaterials with potential of clinical translation. (**A**) A patient case of 3D-printed polymeric ‘clear’ aligners. Reproduced from Ref. [197] with permission of Oxford University Press, © 2022. (**B**) A gastrointestinal (GI) patch for sutureless repair of gastrointestinal defects. Adapted from Ref. [198] with permission of the American Association for the Advancement of Science, © 2022.

Here, we would like to introduce some advanced polymeric materials which have recently been commercialized or have great potential for clinical translation here. In the past years, patients can only choose metal braces with alloy wires for orthodontic treatment, which brought an unfavorable influence on patients during the treatment. Modern orthodontic treatment has adopted polymer as the clear aligner film material for invisible orthodontics. Ding *et al*. [197] reported a polymeric clear aligner fabricated by molding polyurethane films on a 3D-printed dental model (Fig. 4A). The 3D-printed clear aligner offered high precision at each stage of orthodontic treatment to achieve a successful result.

Polymeric materials for tissue adhesion have recently emerged in clinical surgical repair. For example, Zhao *et al*. [198] developed an off-the-shelf adhesive patch consisting of two layers—the top layer was composed of hydrophilic polyurethane for tissue-matching and robust mechanical properties, and the bottom layer was the network between the covalently crosslinked poly(acrylic acid) *N*-hydroxysuccinimide (NHS) ester for adhesion and the physically crosslinked poly(vinyl alcohol) for mechanical reinforcement (Fig. 4B). When contacted with the gastrointestinal defects, the carboxylic acid group in the network allowed the rapid absorption of the interfacial water, and the NHS ester group facilitated robust adhesion to the tissue via covalent crosslinking via imide bonds. Such an adhesive patch could be used for sutureless repair of a gastrointestinal defect. Different from other tissue adhesives and sealants, this adhesive patch achieves rapid and tough adhesion without any external devices.

The adhesive polymeric materials can also be used in some emergency situations like uncontrolled bleeding. Yu *et al*. [199] developed an injectable hemostatic hydrogel that could quickly control blood loss and allow an on-demand dissolution. Such a hemostatic hydrogel was formed *in situ* by blending 4-arm PEG crosslinker modified with thioester linkages and terminated with aldehyde groups and poly(ethylene imine) with adipic dihydrazide. It is amazing that the chemical hydrogel was able to be dissolved via adding a biocompatible L-cysteine methyl ester solution for breaking the crosslinker.

### Bio-derived materials

In light of regenerative medicine, bio-derived materials refer to special composite materials using directly a structural tissue or more frequently acellular matrix (ACM) [200–202]. ACM can be divided into tissue ACM, cell ACM and organ ACM with the first one predominant at the moment. Liang *et al*. [203] studied the difference of autogenous, allograft and artificial bone substitutes on bone regeneration, and suggested that the allogenic bone graft has relatively poor bone repair ability compared with autogenous and artificial bone substitutes probably due to the immunotoxicological reaction. This implies the immunogenicity of allogenic cells, and thus the necessity of removal of those cells.

Decellularization is used for acquiring the extracellular matrix (ECM) structure without residue cells and thus with a reduced immunogenicity [204, 205]. The concept of decellularization came up in 1948 [206], and it has got to be the most widely used approach to remove immunogenic substances. The decellularization methods include physical, chemical and enzyme or their combination [207]. It is worth to mention that decellularization causes more or less disruption of the initial ECM structure, which could be minimized but cannot be completely avoided. Trypsin is an effective decellularization enzyme that can separate cells from structural proteins. While the treatment is useful for preparation of a decellularized vascular graft, it leads to decrease of the strength of the vessel and is adverse to the resistance to blood pressure. Crosslinking is employed to improve mechanical properties of natural biomaterials including physical methods and chemical methods. It is reported that photooxidation crosslinking could significantly increase the residual strength of decellularized vessels, sometimes better than glutaraldehyde (GA) crosslinking [208]. Very recently, Ding’s group put forward a biosurfactant-containing two-step decellularization strategy to modify the biomacromolecular network of bovine pericardium crosslinked with GA, and the resultant bioprosthetic heart valve (BHV) exhibited potent anti-calcification performance according to their implantation experiments [209].

ECM itself is an important natural biomaterial composed of collagens and other molecules that forms a fibrous matrix. Collagen has about 28 subtypes [210], and thus various types of collagens exist in ECMs, depending on the tissues, including dermis, small intestine, heart valves and urinary bladder etc. [211], and further influence the functions of ECM [212]. Small intestine submucosa (SIS) is an ACM biomaterial isolated from the submucosal layer of porcine jejunum [213]. It is composed of several collagens and growth factors, including vascular endothelial growth factor (VEGF), transforming growth factor, basic fibroblast growth factor, etc. SIS is usually used to coat other synthetic scaffolds to improve biocompatibility [214] or used as a biologic patch for tissue repair [215]. Xie *et al*. [216] prepared a polyurethane/SIS hydrogel for endoscopic submucosal dissection-induced ulcer healing.

In addition to the ECMs obtained from animal tissues, cell-derived ECMs are also an alternative material for tissue engineering. For example, Wang *et al*. [217] seeded cardiac fibroblasts on a silk fibroin scaffold. After 10 days of culture, they decellularized the scaffold to obtain the ECM components with myocardial-like properties. Compared to the ECMs from tissues, cell-derived ECMs require easier decellularization because the relatively simple structure will not require complicated dissection and isolation [218]. But the process of decellularization is still a bottleneck of large-scale clinical application of ECMs, which need to be broken for ramping up of production.

It seems worthy of noting that decellularization can sometimes not fully eliminate the immunogenicity. The deleterious immunogenic effects of bio-derived materials have been recognized. Niemann *et al*. [219] transplanted decellularized heart valves that were obtained from sheep (xenogeneic), pigs (allogeneic) and the pigs deficient for the major xenoantigen into the pigs deficient for the galactosyltransferase gene. The xenogeneic sheep-derived heart valve exhibited a strong immune reaction, while the other two allogenic heart valves induced only mild reactions, indicating that decellularization could not sufficiently reduce the immunogenicity of xenogeneic implants.

## Applications of biomaterials in tissue regeneration

Biomaterial science and engineering have been much developed during the latest decade, and the ultimate aim of biomaterial development is the clinical application [220]. Biomaterial scientists and engineers are encouraged to design their materials to meet the requirements from the doctors and patients. Autograft is in most of cases the gold standard for the treatment of tissue defect. However, the use of an autograft generates self-damage. Allografts also face problems, including shortage of donors, heavy cost and risk of immune rejection. Therefore, the development of advanced biomaterials that outperform autograft and allograft can cater to the demand for tissue regeneration. This section will introduce biomedical materials according to their application aspects.

### Bone tissue regeneration

The design of a biomaterial for bone regeneration has evolved from simply fixing by an inert material toward developing a bioactive material that is potentially capable of facilitating the regeneration process. For example, the materials that enable the balance between osteoclast-mediated bone resorption and osteoblast-mediated bone formation to be restored could treat osteoporosis [221].

**Figure 5.**
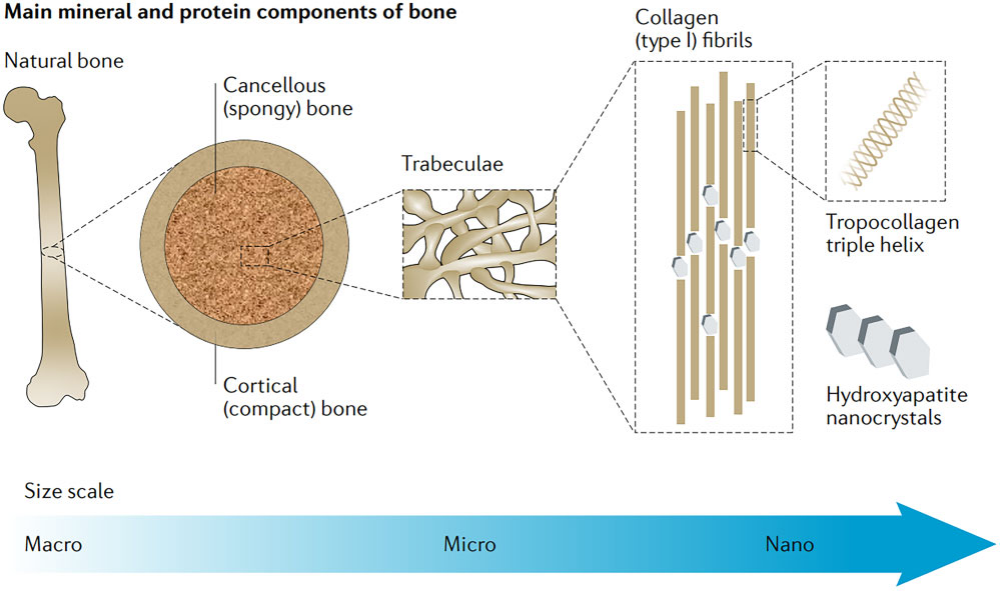
Hierarchical structure of the natural bone to stimulate the design of bone-regenerated biomaterials. The nature bone is composed of hierarchically arranged collagen fibrils and inorganic minerals, which can be mimicked to some extents in fabrication of 3D scaffolds using polymer-ceramic nanocomposites. Reproduced from Ref. [222] with permission of Springer Nature, © 2020.

Natural bone has a hierarchical structure and is composed of several natural ‘materials’. Recently, Mikos *et al*. [222] have well reviewed the components of natural bone and suggested the candidate materials for bone tissue regeneration (Fig. 5). Ceramics, polymers and metals are all the candidate materials to support bone regeneration. Typically, ceramics stimulate mineralization via mimicking the inorganic portion of natural bone [223, 224], polymers work as the bone ECM via mimicking crosslinking of collagen fibrils [225, 226], and metals provide the mechanical support sometimes [227].

The regenerative biomaterials treated for bone tissue usually require a strongly supportive structure [228]. Suitable elastic modulus is an important factor of bone biomaterials. Most metallic materials have higher elastic modulus than natural bones [229]. The mismatch of elastic modulus between these implants and natural bones leads to stress shielding. Stress shielding occurs when an implant bears the majority of loading forces rather than the surrounding bone, which results in the reduced bone density [230]. PEEK is a high-performance semicrystalline thermoplastic polymer with an elastic modulus similar to that of a natural bone and has been approved by FDA as bone implantation. However, PEEK is chemically inert and suffers poor integration with surrounding bone tissues. Therefore, modification of PEEK to improve the bioactivity has been increasingly employed. For example, one has tried to introduce bioactive metals such as strontium, or incorporate hydroxyapatite into PEEK to stimulate cell differentiation [231, 232]. Of course, the corresponding modification should be carried out with the prerequisite not to harm the inherent excellent mechanical strength and chemical stability of PEEK and other pertinent bulk materials.

As biodegradable bone scaffolds are concerned, they are gradually weaker than native bones. Nevertheless, the increased mechanical support provided by new mineralized tissue deposition can offset the material degradation to some extents. Therefore, tuning the rate of scaffold degradation to be synchronized with the rate of new bone deposition is of critical importance for bone regeneration. It is desired that a biodegradable scaffold exhibits little degradation at the initial bone regeneration stage to ensure the sufficient mechanical support and significant degradation at the late stage to leave space for ECM secreted by cells.

Sufficient nutrients and oxygen are dependent on the angiogenesis in tissue regeneration, which are supposed to be a prerequisite for blood flowing to the damaged area within an implant [233]. Restoring the microvascular circulation of an implant is challenging for tissue engineering [234]. For bone regeneration, the ideal scaffold is recognized to have a porous structure which allows for restoring the microvascular circulation and formation of new bone [235, 236]. Therefore, the design of an appropriate porous structure is adopted in bone scaffolds [237, 238]. Apart from this, researchers have made efforts on embedding bioactive substances into implants to accelerate bone formation, such as exogenous growth factors [239–241], peptides [242] and drugs [243].

In addition to bioactive substances, cells like ECs, MSCs, osteoblasts and preosteoclasts can induce angiogenesis. Therefore, attention has been paid to leverage biomaterials to load cells that are capable of vascular reconstruction [244]. An appropriate biodegradable porous scaffold can mimic the ECM for cell attachment, proliferation and differentiation of cells in bone tissue engineering [245]. Accumulated studies have revealed that the implanted biomaterials can play an important role in the regulation of biological activity of host cells [246–248]. Scientists thereby began to seek an approach that enables biomaterials to stimulate host cells to function in angiogenesis. Liu *et al*. [249] tried to use a biomaterial to reconstruct the microvascular network in ischemia for angiogenesis. They previously developed a semisynthetic sulfated chitosan (SCS) with high affinity for VEGF of the sulfated polysaccharide. This time, they coated a gelatin sponge with SCS and implanted it in a mouse model of hind limb ischemia to promote the blood perfusion and angiogenesis. They found that sulfated polysaccharides induced angiogenesis in ischemia via guiding anti-inflammatory macrophages (M2 phenotype) to secret more endogenous VEGF. This work represents a typical design of the future tissue induction biomaterials.

### Cartilage tissue regeneration

Damage of articular cartilage is significant in an elder society. The current regeneration approaches of articular cartilage mainly focus on cell-based therapies of chondrocytes or MSCs [250, 251]. Chondrocytes are the only cell type in cartilage. They show a gradient in density and morphology along the depth of articular cartilage [252]. Chondrocyte transplantation has been applied in clinic [253]. However, utility of autologous chondrocytes as seeding cells faces the challenges of limited donor supply and risk of dedifferentiation, where chondrocytes are gradually converted to fibroblast-like cells leading to failure of chondrogenesis [254]. Chondrocytes were derived from MSCs [255]. MSCs are capable of generating chondrocytes, osteoblasts, adipocytes and myoblasts under specific culture conditions, which makes them an alternative cell type for articular cartilage repair [256, 257]. An appropriate biomaterial can assist MSCs in ramping up the potential of chondrogenic differentiation via mimicking available microenvironment [258–261].

Bio-derived ECM scaffolds have optimistic effects on the differentiation of MSCs, but strongly depend on the original source. The age of the donor of cartilage ECM influences the efficacy of chondrogenesis of MSCs. Among the cartilage ECMs from newborn, juvenile and adult rabbits, the newborn ECM promoted the most chondrogenesis of MSCs but led to matrix calcification, which severely limit the application [262]. Besides, collagen I has been proved to induce the chondrogenic differentiation of MSCs, while avoiding dedifferentiation of chondrocytes turns out to be a challenge sometimes [263]. They suggested that physically crosslinked collagen scaffold may contract under the action of cellular activity, because the weak hydrogen bonding may readily to unbind when subjected to the reaction of actin. The contraction is a mechanical stimuli to MSCs, which may lead to the potential dedifferentiation. Therefore, Guo *et al*. [264] used a photo-crosslinking approach to enable a collagen hydrogel to restrict the contraction and maintain the chondrocyte phenotype without dedifferentiation.

**Figure 6.**
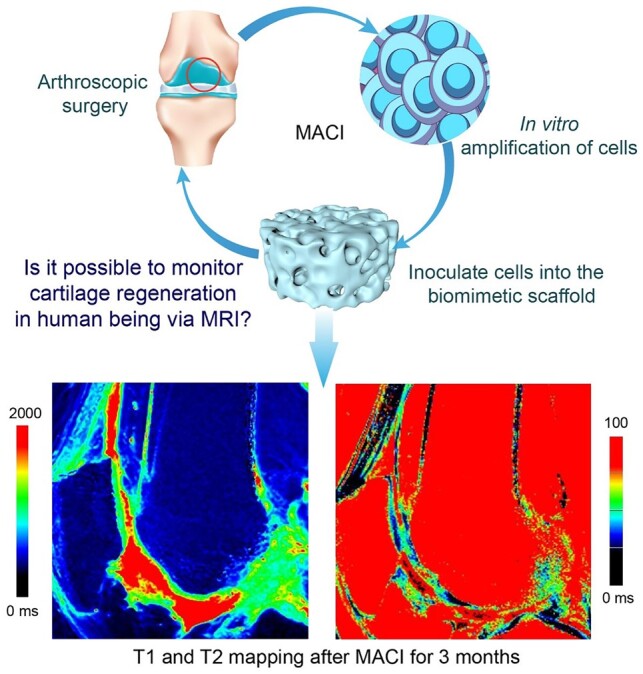
Noninvasive clinical evaluation of cartilage regeneration. Patients who experienced arthroscopic surgery for collecting autologous chondrocytes would accept matrix-induced autologous chondrocyte implantation (MACI) for cartilage regeneration, and the MRI is a feasible semi-quantitative yet noninvasive way for clinical follow-up. T1 (left) is the spin–lattice relaxation time with respect to longitudinal relaxation; T2 (right) is the spin–spin relaxation time with respect to transverse relaxation. Adapted from Ref. [265] with permission of Oxford University Press, © 2021.

A noninvasive approach for the evaluation of regenerated cartilage is crucial for clinical follow-up. Xu *et al*. [265] followed 18 patients who experienced matrix-induced autologous chondrocyte implantation, and analyzed their MRI results (Fig. 6). They found that the collagen network became matured and the proteoglycan content increased in the regenerated cartilage through MRI imaging, indicating that MRI is an excellent way for the noninvasive follow-up of cartilage regeneration. Based on these research and verification, the Chinese team suggested an assessment standard, and in September, 2022, the International Standard Organization (ISO) approved ‘Tissue Engineered Medical Products—MRI Evaluation of Cartilage—Part 1: Clinical Evaluation of Regenerative Knee Articular Cartilage Using Delayed Gadolinium-Enhanced MRI of the Cartilage (dGEMRIC) and T2 Mapping’ as ISO/TS 24560-1:2022.

Cartilage has limited spontaneous repair ability due to its condensed construction and devoid of blood vessels [266, 267]. Osteochondral defect in clinic involves the defects of articular cartilage and subchondral bone. These two layers have different mechanical strengths and biological lineages, and require distinct repairing approaches [268]. Therefore, some bilayered scaffolds are designed to simultaneously regenerate articular cartilage and subchondral bone [269, 270]. The upper layer of a bilayered scaffold can be made of a relatively soft polymer material to match the cartilage regeneration, while the bottom layer can be equipped with a bioactive ceramic to regenerate the subchondral bone. Besides, the bilayered scaffolds have another advantage that the defected cartilage can easily obtain nutrition from the regenerated subchondral bone [271].

### Biomaterials for cardiovascular repair

Cardiovascular diseases are one of the threats to human health with high morbidity and mortality. The treatment of cardiovascular diseases much relies on biomaterials, including vascular stents, grafts, occluders, heart valves and etc. Implants for cardiovascular repair are generally made of metallic, polymeric and bio-derived materials.

Owing to the appropriate mechanical strength, metallic stents have been used in clinic. Bare metal stents are the first-generation for the percutaneous coronary intervention. However, the in-stent restenosis of a bare metal stent has limited their clinical application [272]. Therefore, the second-generation stents were invented to use a drug coating to reduce restenosis via limiting the proliferation of associated cells [273].

**Figure 7.**
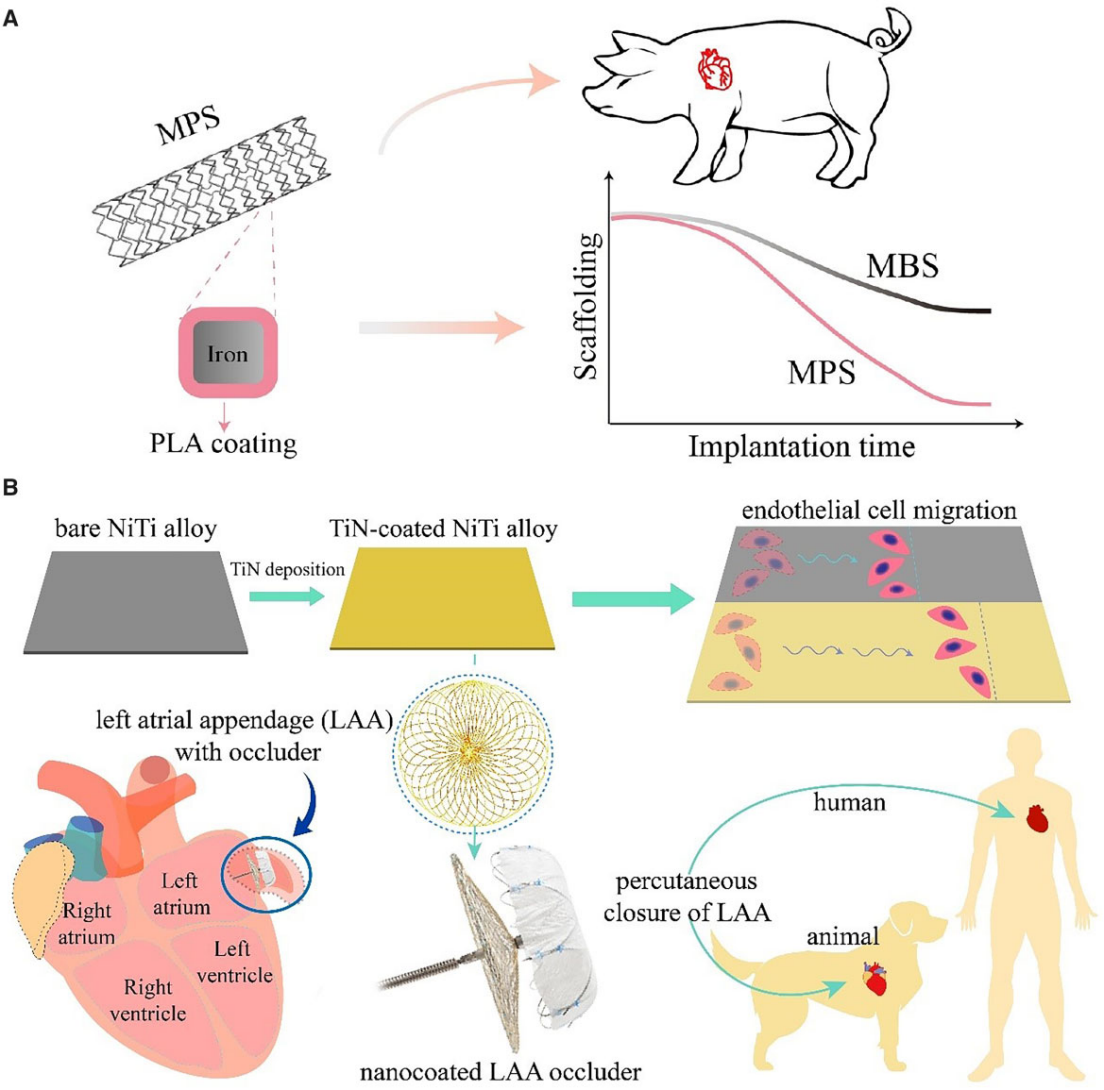
Implants for cardiovascular repair. (**A**) Using a polymer coating to accelerate the corrosion of iron by fabrication of a metal–polymer composite stent (MPS). Reproduced from Ref. [275] with permission of Elsevier, © 2021. (**B**) Surface modification of nitinol to enhance cell migration and the corresponding left atrial appendage (LAA) occluder with nanocoating. Reproduced from Ref. [58] with permission of American Chemical Society, © 2020.

Biodegradable or bioresorbable stents is the newest-generation ones that enable to tackle the problems which the traditional bare metal stents and drug-eluting stents are faced with. Along with the disappearance of a bioresorbable stent, the regenerated vessel fully sustains natural blood flow of the vasoconstrictive characteristic [274]. Bioresorbable stents are usually made of corrosive metal or degradable polymers. The undesirable degradation behavior of metallic stents may become an impediment for cardiovascular regeneration. For example, the iron stents can remain uncorroded after vascular remodeling (usually 3–6 months), and is thus unsuitable as an ideal biodegradable stent unless further modifications. Utilizing composite materials can enhance the properties of metallic materials. Despite painting metallic materials with organic materials is a common method to achieve corrosion protection, Ding *et al*. [275] suggested an interesting method and confirmed that coating PLA on an iron stent achieved complete *in vivo* corrosion of the iron stent in 3–6 months (Fig. 7A). The acceleration of iron corrosion by this polymer coating was partially explained by the decreasing local pH along with PLA hydrolysis [47]. The endothelial coverage on an MPS was found to be better than that on a permanent stent after implantation in a rabbit model, indicating the lower risks of stent thrombosis [276]. Very recently, they found that serum, especially the albumin can reduce the free radicals generated during iron corrosion [277].

A full-chain research covers many aspects from fundamental studies to translational efforts. In the biomaterial field, a full-chain research is a cooperative one from bench to beside. For example, a nano-coated medical device for closure of left atrial appendage has recently been developed, which is associated with rapid endothelialization following interventional treatment [58]. Endothelialization is critical for implanted cardiovascular devices, in which adhesion, migration and proliferation of ECs take place on the implanted materials [278]. The vascular endothelium has antithrombotic properties via generating NO, prostacyclin and etc., and plays the role of the barrier between the blood and vessel wall [279]. The intravascular procedures inevitably cause vascular injury and result in the damage of vascular endothelium, which further lead to thrombosis and restenosis. Therefore, researchers have explored a biomaterial that can achieve rapid endothelialization after implantation via designing an appropriate cell-material interaction. Ding *et al*. [58] found that surface modification of TiN-coated NiTi ally could significantly enhance the cell migration *in vitro* and achieve a rapid endothelialization *in vivo* (Fig. 7B). Cheng *et al*. [280] designed an exosome-eluting stent, which accelerated re-endothelialization via releasing ROS after implantation.

Recently, transcatheter aortic valve implantation has been a hot issue in the clinical treatment of heart valve disease. Patients with demand of implantation of cardiac valves can select mechanical or xenogeneic bioprosthetic valves, and the BHVs are the main artificial heart valves. Compared to mechanical valves, the bio-derived BHVs exhibit better hemodynamic performance, and do not need lifelong anticoagulation. After decellularization, the xenogeneic BHVs experience chemical crosslinking usually by GA treatment [281]. However, such valves must be stored in a GA solution for preservation and sterilizing. Dissolved GA is a potential mutagen and can induce significant cytotoxic and mutagenic effects in mouse lymphoma cells [282]. In recent years, the advent of a dry valve has been paid attention to. Wang *et al*. [283] fabricated such a non-GA-crosslinked dry valve, which was crosslinked by the combination of carbodiimide and polyphenol. In principle, the surgeons can immediately use the dry valve from a sterilized bag, which save the preoperative preparation time.

### 3D bioprinting of both biomaterials and cells for tissue regeneration

Tissue engineering and tissue regeneration reply on porous scaffolds as usual. While many scaffolding techniques have been developed and the mechanical properties of the porous scaffolds have been investigated [284–287], the porogening and shaping need different procedures until the emergence of 3D printing, where both the internal pore and external shape of a porous scaffold can be controlled simultaneously by one core technique.

The 3D printing is attractive because of also its ability to deal with raw materials and cells together through the so-called bioink. Many cells especially stem cells have the great potential in tissue regeneration with the ability to self-renew and differentiate into different cell types in response to the environmental cues [288–290]. However, the major challenge of transplanting cells into human bodies is the low viability and efficacy. Nevertheless, an appropriate biomaterial can act as a delivery vehicle and an artificial matrix to provide the loaded cells with both a structural support and a microenvironment for cells living and differentiation [291, 292]. A hydrogel holds a great promise in this field, not only because its compatibility but also for its printability, and some researchers have employed hydrogels to study cell behaviors or address an unmet need [293–295]. For example, Liu *et al*. [296] fabricated a 3D self-assembling peptide hydrogel to study the stem cell fate in a biomimetic extracellular microenvironment. Some advanced techniques have been developed to use hydrogels to print 3D cell-laden constructs via 3D bioprinting [297, 298]. Specifically, isolated cells were suspended in hydrogels, and the bioink was subsequently extruded into continuous fibers to form a cell-loaded 3D scaffold [299–301].

**Figure 8.**
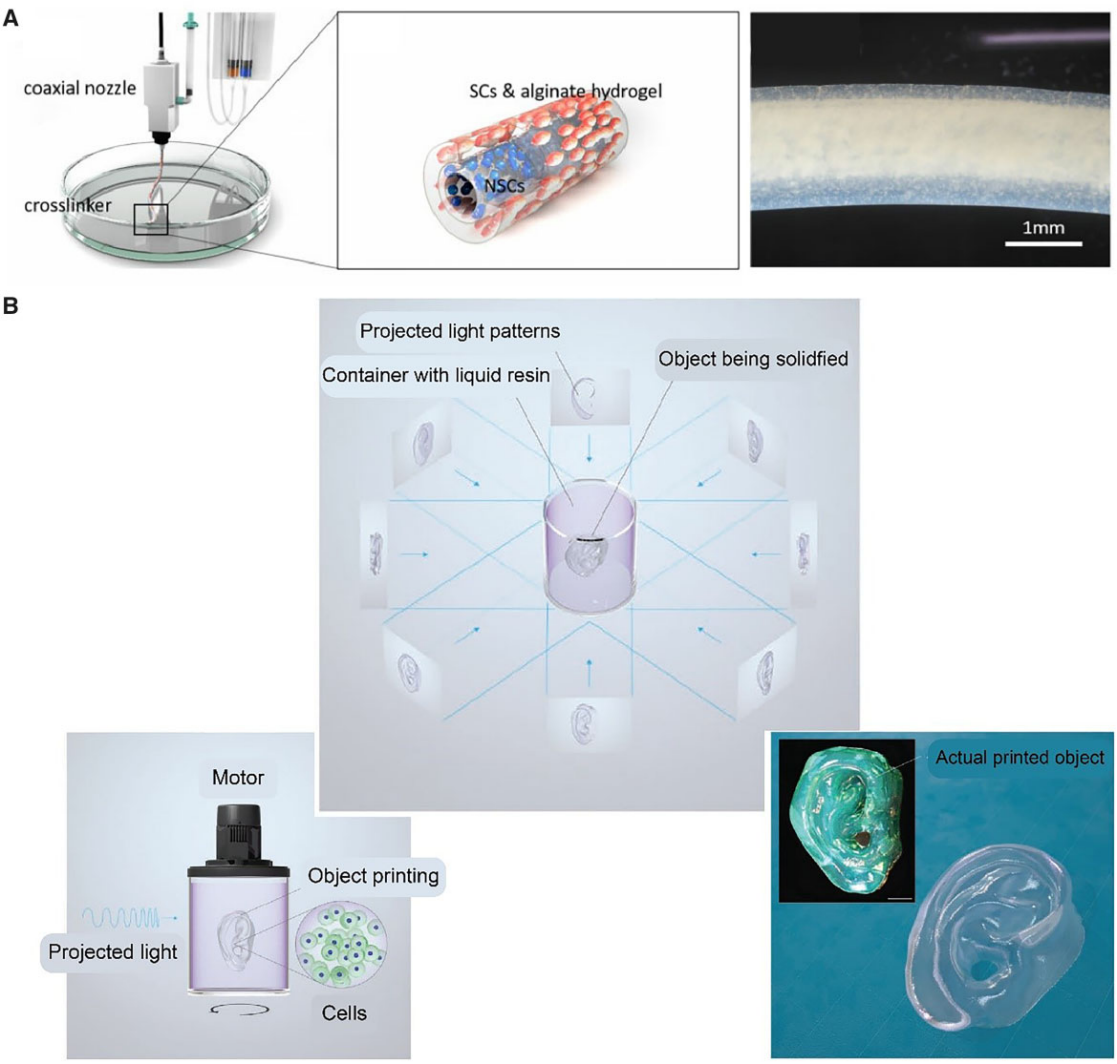
Biomaterials for being printed together with cells. (**A**) Schwann cell (SC)-neural stem cell (NSC) core–shell alginate hydrogel fibers fabricated via coaxial extrusion. Reproduced from Ref. [303] with permission of Oxford University Press, © 2021. (**B**) Volumetric bioprinting based on light projection using cell-laden gelMA PBS solution as bioink. Adapted from Ref. [304] with permission of Wiley-VCH, © 2019.

The 3D-printed ECM can afford a complex microenvironment for loaded cells via integrating biophysical and biochemical cues. Huang and Fu *et al*. [302] used an alginate/gelatin hydrogel to print a scaffold for MSCs with 3D bioprinting. The 3D-printed scaffold directed the differentiation of loaded MSCs and ultimately guided the formation and function of glandular tissue. Xu *et al*. [303] also used an extrusion method to print a cell-loaded alginate hydrogel for the treatment of spinal cord injury (Fig. 8A). Interestingly, they printed a scaffold with a core-shell structure composed of Schwann cells and neural stem cells in a coaxial extrusion to mimic nerve fibers. They observed the positive effect on the differentiation of neural stem cells in the co-culture model. The aforementioned conventional 3D bioprinting generates a 3D shaped biomaterial by layer-by-layer precise positioning *in vitro*, and then it could be implanted *in vivo* by surgery.

The layer-by-layer dogma was broken recently along with the emergence of an advanced bioprinting strategy. Different from the limited printing velocity of layer-by-layer deposition of the conventional 3D bioprinting, a volumetric bioprinting based on visible light projection was developed. Moser and Levato *et al*. [304] invented volumetric bioprinting to fabricate a cell-laden human auricle model within seconds. Specifically, 3D light dose distribution was deposited into a rotating cylindrical container containing a gelMA phosphate buffer saline solution with photoinitiator to permit the spatially selective polymerization (Fig. 8B). The bioprinting of both materials and cells is still in rapid progress.

### Wound healing

Wound dressing materials have been developed with some exciting results [305–307]. A hydrogel is particularly useful for wound healing owing to its tunable mechanical stability, high compatibility and drug delivery capability [308]. Peptide hydrogels are based on self-assembly and has been paid much attention to [309]. One of the concerns in wound healing is to prevent bacterial infections [310, 311]. Because infection leads to many severe problems such as chronic wound, researchers tried to employ some biomaterials of photothermal conversion ability, such as iron oxide and PDA, to endow wound dressing with excellent antibacterial effect [312, 313]. Zheng *et al*. [314] used bacterial cellulose, a natural polymer secreted by bacteria, to fabricate a wound dressing combined with tannic acid and magnesium chloride for anti-biofilm in chronic wounds.

At least three aspects should be considered into the design of a wound dressing material—inflammation, proliferation and remodeling [315, 316]. For example, inhibiting the generation of pro-inflammatory cytokines is an approach to accelerate wound healing [317, 318]. Since ROS could lead to inflammatory responses in the early stage, scavenging ROS has been considered in wound dressing development. Gu *et al*. [319] fabricated a hybrid hydrogel of superabsorbent poly(acrylic acid) and antioxidant poly(ester amide) to absorb the exudate to scavenge ROS for wound healing. Gong *et al*. [320] induced cerium oxide nanorods, a kind of ROS scavenger, into a thermosensitive hydrogel via Schiff base reaction to enhance the wound healing. For the patients with extensive skin burns, skin grafting is a mainstream treatment in clinic. But considering that an autologous skin graft is limited, developing skin substitutes such as acellular dermal matrix may be a way to meet the clinical demand [321]. Human amnion shows promising clinical benefit in wound healing owing to the regenerated activities of amniotic membrane and the inside viable human amnion epithelial cells [322, 323]. Therefore, the technologies that could retain the viable cells and the biologically active components are the impetus of a widespread use of amnion.

### Biomaterials for medical cosmetology

With the development of the world economics, people care more aesthetics, and the global cosmetics market has reached hundred billions of US dollars. While medical cosmetology is an important and hot field, little corresponding literature can be found in the database. Biomaterials have been applied in the medical cosmetology for many years, such as filler materials and cosmetics. Filler materials can be injected in facial tissue to achieve facial rejuvenation, which requires safe and easy-to-operate biomaterials [324]. A few polymer materials have been developed for facial injection, such as polyacrylamide, hyaluronic acid, PLA and PLGA. Recently, it has been reported that PLA microspheres stimulate collagen regeneration when used as aesthetic materials such as dermal fillers [325].

In the plastic surgery, the use of allografts is associated with a risk of disease transmission from the donor and the use of autografts may result in additional morbidity associated with healing of the donor site. For example, rhinoplasty patients usually chose an autologous graft in clinic at present, but suffer severe pain and morbidity at the donor site. Ao *et al*. [326] evaluated an xenogenic decellularized costal cartilage graft used as rhinoplasty prostheses, which may emerge as a promising alternative material for plastic surgery.

**Figure 9.**
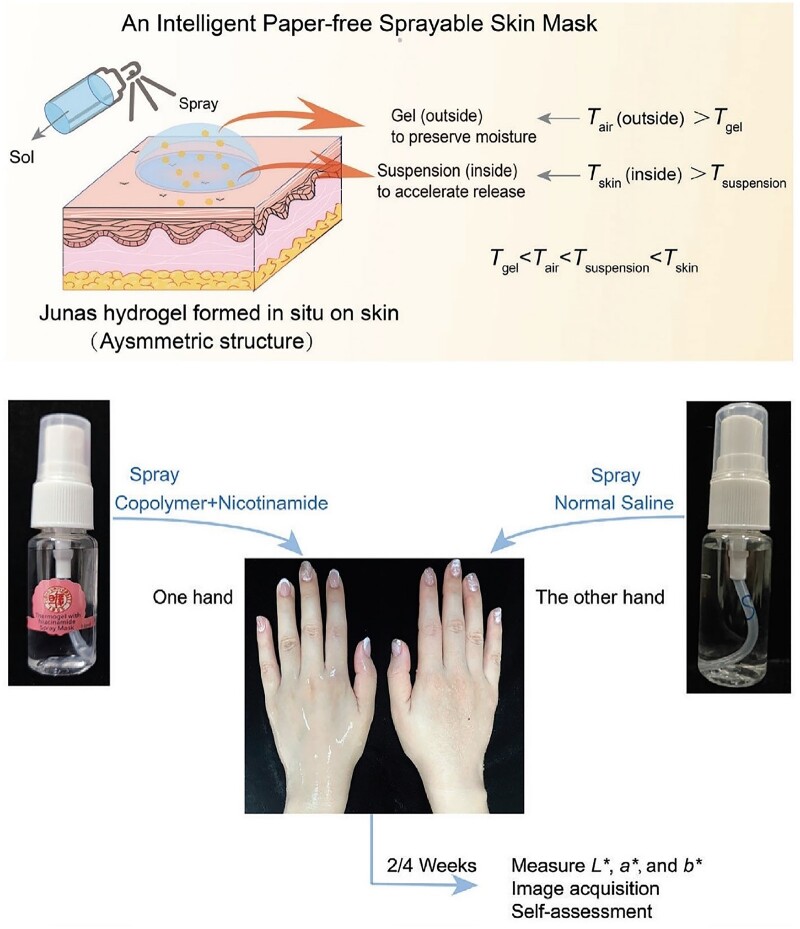
Biomaterials for medical cosmetology: an intelligent paper-free sprayable skin mask based on environmental-friendly thermogel and its first clinical research. Reproduced from Ref. [327] with permission of Wiley-VCH, © 2022.

Besides plastic surgery, biomaterials can be applied in cosmetics and aesthetic medicine. Even some exciting smart materials have recently been reported. As shown in Fig. 9, an intelligent sprayable mask could spontaneously form a ‘Janus’ hydrogel, in which the outside contacting the air is Gel, and the inside contacting the skin is Suspension [327]. Such a paper-free mask was fabricated by environmentally friendly thermogel composed of PLGA-PEG-PLGA block copolymers. Previously, PLGA-PEG-PLGA block copolymers have been tried in drug delivery and submucosal fluid cushion of endoscopic submucosal dissection [328, 329]. Ding’s group carried out the first-in-human study of this polymer on skin. Their results confirmed the safety and efficacy of this synthetic copolymer, which might have impact to the potential translation of the underlying biodegradable copolymer [327]. Their hydrogel mask promotes the release of active substances and keeps moisture via the asymmetric ‘Janus’ structure. The principle of such an asymmetric structure of thermogel has been revealed in their publication [330]. The aqueous systems of PLGA-PEG-PLGA block copolymers exhibit two phase transitions: sol-gel transition and gel-sol(suspension) transition. While the applications based on the first transition (sol-gel transition) have been applied widely in biomedical formulations, the second transition, namely, gel-to-sol(suspension), has not yet been taken into consideration before. This time, Ding group adjusted both of the two phase-transition temperatures to meet the requirements of ‘*T*_gel_ < *T*_air_ < *T*_suspension_ < *T*_skin_’, and developed a transdermal hydrogel formulation [327, 330]. Here, *T*_gel_ is the sol-gel transition temperature; *T*_air_ is the room temperature; *T*_suspension_ is the gel-sol(suspension) temperature; *T*_body_ is the body temperature.

### Drug/gene delivery systems associated with regenerative medicine

Drug delivery platforms based on biomaterials have revolutionized the treatment outcomes for millions of patients [331–333]. Traditional biomaterials for drug delivery are recognized as the excipient that is able to improve safety, optimize the pharmacokinetic profile, or change the routes of administrations of active pharmaceutical ingredient (API). With the development of pharmaceutical sciences, one can finely tune the release of the encapsulated API, including sustained release and controlled release. Along with the emergence of regenerative biomaterials, researchers have gradually realized that some pharmaceutical excipients can be extended to an intelligent drug delivery [334, 335]. For example, self-assembly of amphiphilic polymers can be employed to significantly improve the bioavailability of hydrophobic drugs [336, 337]; appropriate nanotechnology can lower the adverse effect owing to the accumulation of drug in tumor tissue via passive target etc. [338–340]; prodrug micelles are capable of protecting API via covalently bonding an API to amphiphilic polymers [341, 342]; PEGylation, namely, covalently grafting a PEG polymer to a drug can enhance the therapeutic properties of API by prolongation of the drug circulation in plasma [343]; even simply mixing with PEG can enhance the fraction of the active chemical form of antitumor drugs of the camptothecin family by modification of drug–material interaction [344]; cell-derived biomimetic nanoparticles endow drugs with many features such as active target, low immunogenicity and long circulation time [345–348]; various responsive polymers have been tried to positively affect the controlled release of API [349–351]; scaffolds loaded with drugs or modified with other biologically active moieties have been employed to support tissue regeneration [352–354]. All of these indicate that the modern drug delivery system (DDS) is based on the development of exciting materials to a large extent and the development of an advanced DDS is in turn beneficial for regenerative medicine.

**Figure 10.**
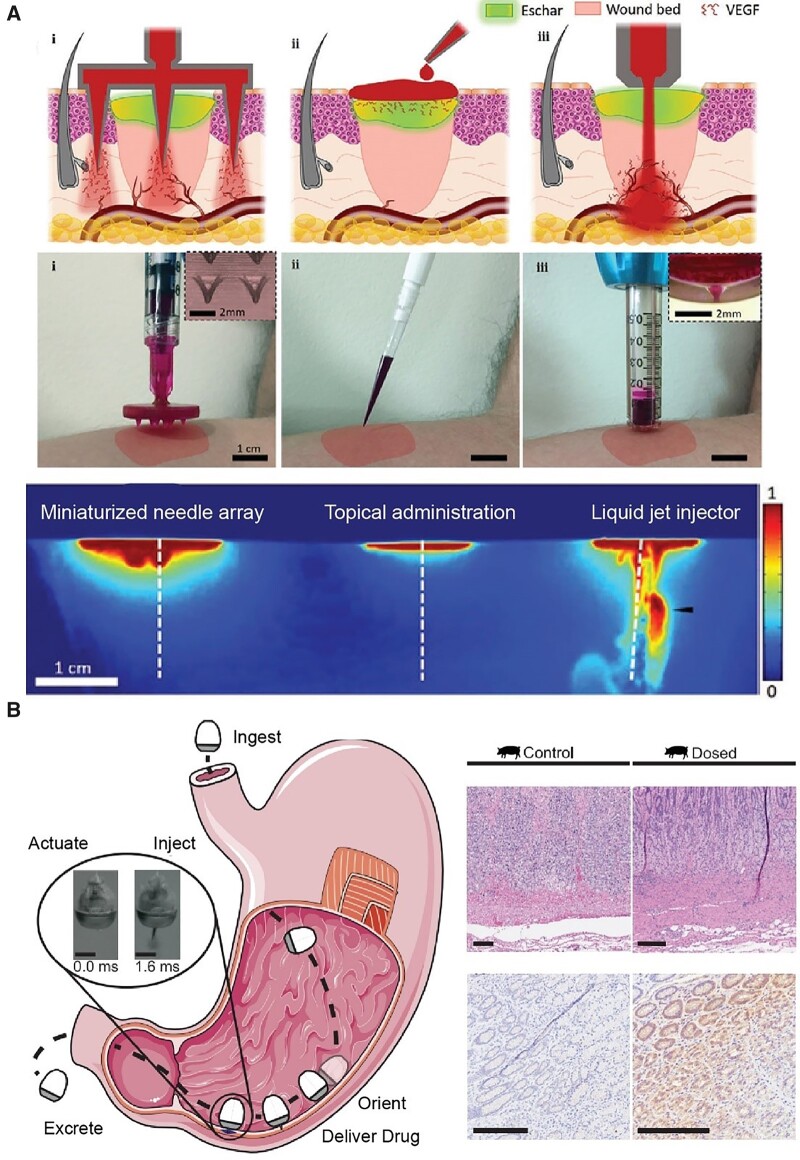
Biomaterials platform for regenerative medicine delivery. (**A**) The drug exhibit spatial distribution between different administrations for wound healing including miniaturized needle array (**i**), topical administration (**ii**) and liquid jet injector (**iii**). Adapted from Ref. [357] with permission of Wiley-VCH, © 2021. (**B**) Orally dosed milli-injector capsules enable nucleic acid delivery to swine stomachs. The right part shows hematoxylin and eosin-stained histology (upper) and immunohistochemistry histology (lower) stained against RNA encoding Cre recombinase enzyme. Scale bars indicate 200 mm. Adapted from Ref. [358] with permission of Elsevier, © 2022.

We further introduce some advanced delivery systems that are based on an innovated route of administration. Microneedle is a promising technology for the transdermal drug delivery, which can avoid the hepatic first pass metabolism and be particularly suitable for skin regeneration [355]. During the wound healing progress, sufficient vascularization can avoid many unexpected events such as hypoxia, poor metabolic support and dysregulated immune response that finally lead to chronic wounds [356]. Delivering VEGF into the wound bed may stimulate proper vascularization of the wound. Peppas, Sinha and Tamayol *et al*. [357] have illustrated the capability of microneedles for deep penetration compared with topical administration. As shown in Fig. 10A, miniaturized needle arrays and liquid jet injectors exhibit better drug spatial distribution in the wound bed compared with topical administration, indicating the importance delivery strategy in wound healing.

A particularly important case to employ biomaterials to enhance pharmaceutics is the development of oral administration [358]. Oral administration of drugs is one of the most popular administration routs for patients, especially for patients with chronic diseases, but used to be regarded inappropriate for biomacromolecular drugs such as proteins, DNA, mRNA owing to their instability in gastrointestinal tract. This ‘impossibility’ has been gradually broken by many distinguished scientists such as Leong et al. [359]. More recently, as shown in Fig. 10B, Langer and Traverso groups have demonstrated an ingestible milli-injector capsule that delivers mRNA through the gastrointestinal tract with high transfection efficiencies [358]. Such a pill could propel a needle to inject the loaded drug into vascularized layers of stomach tissue once self-oriented in gastric submucosa. Naked mRNA can be quickly degraded by extracellular RNases and cannot be internalized due to its electronegativity. Therefore, an mRNA carrier composed of cationic lipid, polymer and peptide were developed for loading and facilitating cellular uptake. They chose branched hybrid poly(β-amino ester) nanoparticles to encapsulate the mRNA and achieved satisfactory transfection efficiency. For delivery of mRNA as well as other nucleic acids, this work gives a brand-new angle of view. Different from the oral formulation, Sumita *et al*. [360] fabricated a gene-activated matrix comprised of atelocollagen, TCP granules and naked-plasmid DNAs encoding microRNA 20a that promotes osteoblast differentiation by inhibiting the negative regulation of osteoblasts. They implanted the gene-activated matrix onto the cranial bone surfaces of rats, and found that bone augmentation was promoted up to 8 weeks after transplantation.

## Biomaterials applied in public health emergency

People from both World Health Organization (WHO) and most of countries have painfully recognized that a public health emergency can cause severe consequences for globe health after experiencing the outbreak of COVID-19, caused by a novel coronavirus named severe acute respiratory syndrome coronavirus 2 (SARS-CoV-2) [361]. Considering the important role of biomaterials in public health emergency, we would like to make a generalized discussion of regenerative biomaterials particularly for the application of biomaterials in COVID-19 pandemic, including virus detection, vaccine enhancement, treatment of infection and post-infection.

### Biomaterials for COVID-19 relevant medical devices

**Figure 11.**
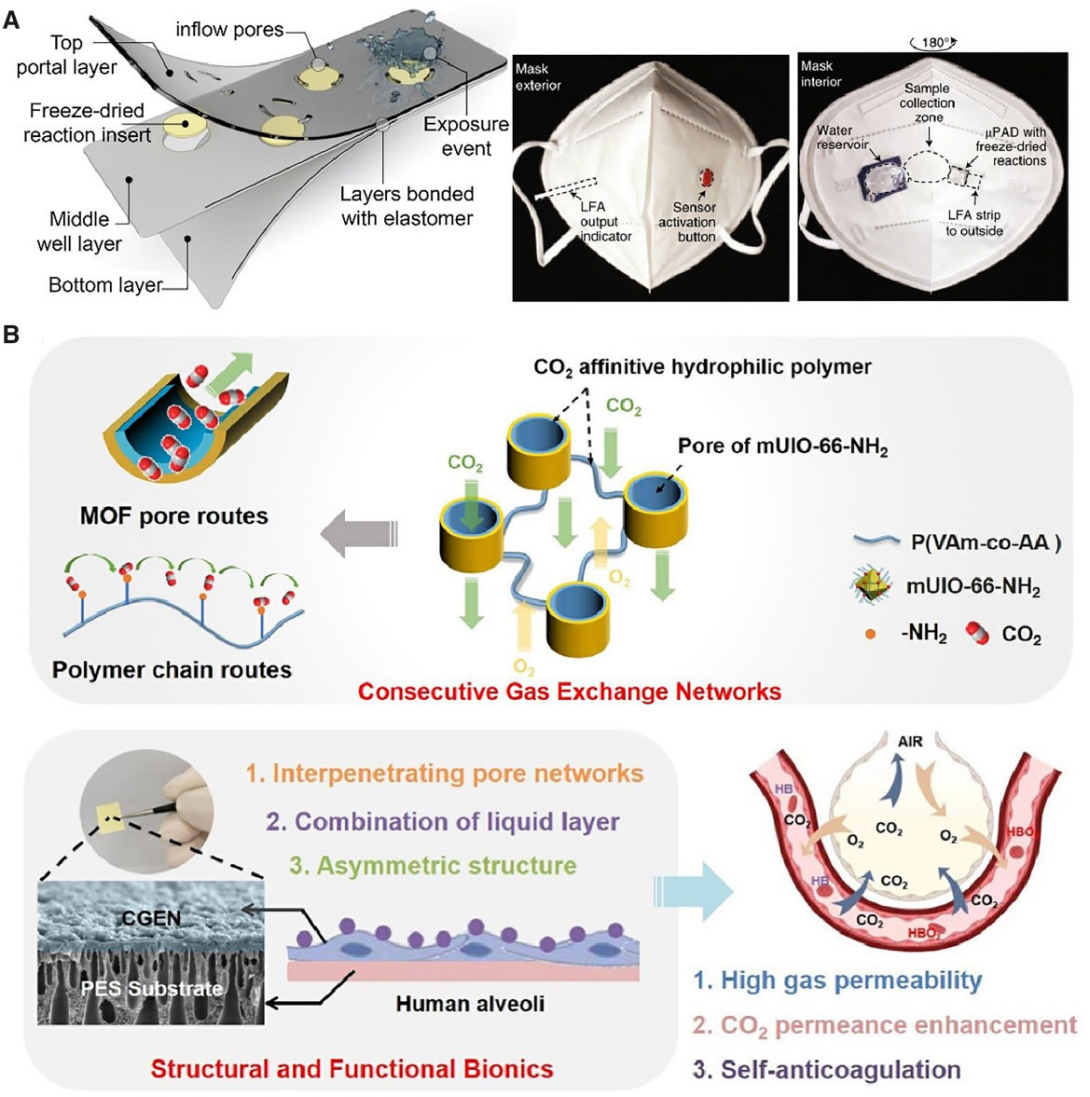
Biomaterials associated with COVID-19. (**A**) A mask equipped with a COVID-19 detected sensor. Adapted from Ref. [364] with permission of Springer Nature, © 2021. (**B**) An anticoagulant biomimetic gas exchange membrane used for ECMO. Reproduced from Ref. [365] with permission of Elsevier, © 2022.

Until now, the detection of COVID-19 still relies on the nucleic acid tests by quantitative reverse transcription–polymerase chain reaction, which requires a few hours of processing for pre-treatment of a sample and DNA amplification by professional staff using specific equipment. Reverse transcription–polymerase chain reaction has been regarded as the gold standard for COVID-19 detection. With the development of modern biomaterials, novel technologies and facilities with high speed, accuracy and sensitivity have emerged for virus detection [362]. Utilizing ultrasensitive biosensors may accomplish the goal to develop a rapid and easy-operated detection method. Wei *et al*. [363] implemented an electromechanical biosensor into an integrated and portable device, which can quickly accomplish the COVID-19 detection. Collins *et al*. [364] developed a synthetic biology sensor that could be embedded in a wearable material for COVID-19 detection. They unified such a sensor into a mask to detect the potential coronavirus, which may exist in the air (Fig. 11A).

Another example of medical devises to deal with COVID-19 is about extracorporeal membrane oxygenation (ECMO) [365]. Timely and effective supports play a key role in the treatment of COVID-19-related acute hypoxemic respiratory [366, 367]. ECMO is a device to realize the outside circulation of body blood for oxygenation on a biomaterial membrane that could passage oxygen and carbon dioxide. The oxygenator membrane is the core component of ECMO, requiring high permeability and minimized risk of gas embolism. The modern oxygenator membrane adopts the hollow fiber membrane to provide an appropriate pore size and a high porosity for gas exchange without blood leakage [368]. Hydrophilic materials may cause the leakage of blood, while hydrophobic materials may lead to undesired protein adsorption and platelet adhesion. So, it is critical to adjust an appropriate hydrophilic/hydrophobic balance of an ECMO membrane.

Thrombosis is the common problem faced by any material contacting with blood. The ECMO materials have large areas to contact with blood, and thus modification of the corresponding material surface is important for ECMO toward its clinical application [369]. Introduction of an anticoagulant coating is an efficient approach to reduce thrombus formation. For example, Medtronic company, an ECMO supplier, adopted grafting heparin onto the material surface [370]. Additionally, generation of a biomimetic membrane interface holds great promise in achieving more efficient gas transmission processes of ECMO. Zhao *et al*. [365] fabricated an anticoagulant biomimetic gas exchange membrane inspired from the structure of the human alveoli. As shown in Fig. 11B, they employed porous MOF particles and CO_2_ affinitive hydrophilic polymer to establish the main gas exchange channels, and used polyethersulfone for mechanical support.

### Biomaterials for COVID-19 relevant vaccines and drugs

In 2022, significant progress in fighting against COVID-19 has been made. For example, a few vaccines have entered into clinic. While traditional vaccine development usually takes several years for antigen selection, preclinical animal studies and clinical studies, the situation of public health emergency, especially COVID-19 pandemic, requires the most rapid development of vaccine [371]. Actually, R & D of a COVID-19 vaccine has been condensed into a few months, which is very challenging [372]. The development of vaccine has evolved from live-attenuated and inactivated vaccine to the adenovirus-vectored vaccine and novel mRNA vaccine. The mRNA vaccines produce the antigen of interest for eliciting potent immunity via mRNA translation, which has opened a new era in vaccinology. Interestingly, before the pandemic, researchers held the view that there was a long way to go before the translation of mRNA vaccines. However, the COVID-19 pandemic gives the chance for mRNA vaccines to accomplish the clinic translation for the first time. One of the biggest advantages of mRNA vaccines is that they can be produced and scaled up within weeks after the identification of target antigen, which is crucial for the situations where an urgent pandemic outbreaks.

**Figure 12.**
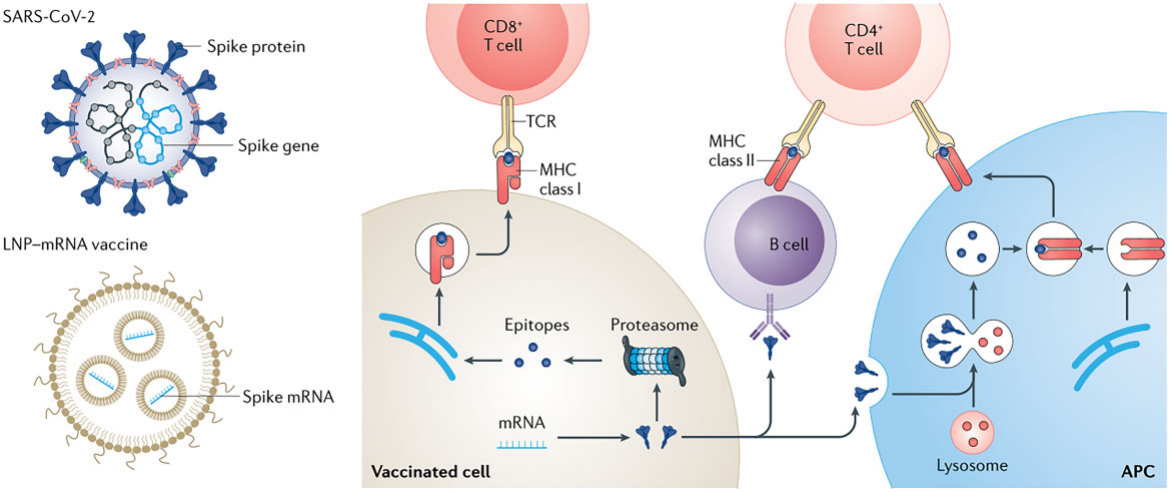
Lipid nanoparticles for COVID-19 mRNA vaccines. Reproduced from Ref. [375] with permission of Springer Nature, © 2021.

The successful translation of mRNA vaccines would not be achieved without the support of an appropriate delivery system [373]. On the one hand, mRNA is a kind of macromolecules, which can only be endocytosed into cells and subsequently be transported into lysosomes for degradation. On the other hand, the natural RNases in the body can rapidly hydrolyze mRNA. Therefore, researchers utilize biomaterials to solve the delivery problem and achieve more potent immune responses. The vehicles for mRNA delivery contain lipid nanoparticles, polymers, peptides and cationic nano-emulsion. Both of the two marketed mRNA vaccines, mRNA-1273 (Moderna) and BNT-162 (BioNtech/Pfizer) use lipid nanoparticles for mRNA delivery [374]. As shown in Fig. 12, COVID-19 mRNA vaccines are formulated with lipid nanoparticle and mRNA encoding spike proteins [375]. After administration, the lipid nanoparticle vector improves the efficacy of cellular uptake and facilitates the endosomal escape of mRNA. After the translation, spike antigens are expressed in the cytoplasm. Some of them are degraded into epitopes, and the others are possessed and delivered by antigen-presenting cells (APCs) for presentation. According to the clinical results, the long-term immunity is still a challenge for vaccine development due to the high mutation of coronavirus, and some advanced biomaterial technology has been tried for the vaccine enhancement [376]. Zeng *et al*. [377] developed a quadrivalent mosaic nanoparticle vaccine that combined four spike proteins, including SARS-CoV-2 prototype and three different variants against COVID-19 infection.

The treatment of COVID-19 mainly depended on antiviral drugs and anti-inflammatory drugs [378–380]. The infection of SARS-CoV-2 started with the engagement between the spike protein of the virus and the angiotensin-converting enzyme-2 (ACE-2) on host cells [381]. Inhibiting this interaction is thus promising for COVID-19 treatment [382, 383]. A polymer able to interact with ACE-2 may impair the infection of COVID-19 [384]. Besides, some carbon-based nanomaterials show promising antiviral activities against RNA viruses, which can be the candidates for COVID-19 treatment [385].

In some severe clinical cases, patients with SARS-CoV-2 suffered excessive inflammation [386], respiratory failure [387], coagulopathy [388, 389] and probable death, which implied the potential risks of organ trauma. Fortunately, biomaterials have an impactful role in the tissue regeneration of post-infection of COVID-19. The strategy based on biomaterials for COVID-19 relevant tissue regeneration is similar to the drug delivery aforementioned in this review. For example, MSCs have the potential to regenerate lung damage, which may offer a therapeutic option for patients with severe or critical COVID-19 [390]. Besides, MSC-derived exosomes also exhibit anti-inflammatory efficacy and the ability to induce tissue regeneration [391].

## Cell–biomaterial interactions

Understanding of interactions between biomaterials and cells is, as a very fundamental study, vital for the development of the next generation biomaterials. On the one hand, cell adhesion, migration and differentiation etc. on biomaterials are investigated mainly *in vitro* in well-defined systems [392–397]. On the other hand, implantable biomaterials are inevitably in contact with immune cells, which gives rise to host responses [398]. The immune cells can influence the long-term regeneration outcomes of biomaterials via mediating the inflammatory responses. It turns out that immunomodulatory biomaterials promote tissue regeneration or treat disease via manipulating immune cells [399]. In this section, we mainly discuss the cell behaviors regulated by well-controlled patterned surfaces *in vitro* and the immunomodulation effects of cell-biomaterial interactions *in vivo*.

### Fundamental research to reveal the material factors to regulate cell behaviors

Cells exhibit various behaviors on material surfaces such as adhesion, migration, communication, proliferation and differentiation. Various approaches have been employed to improve cell adhesion [400–402]. For example, PDA coating has been widely used to enhance cell adhesion by mediating a bioactive material to a substrate. Interestingly, Ding group has very recently reported that the total viability of cells on the PDA coating exhibits time dependence [403]. Besides, RGD peptide is the most frequent sequence for stimulating cell adhesion [404, 405].

**Figure 13.**
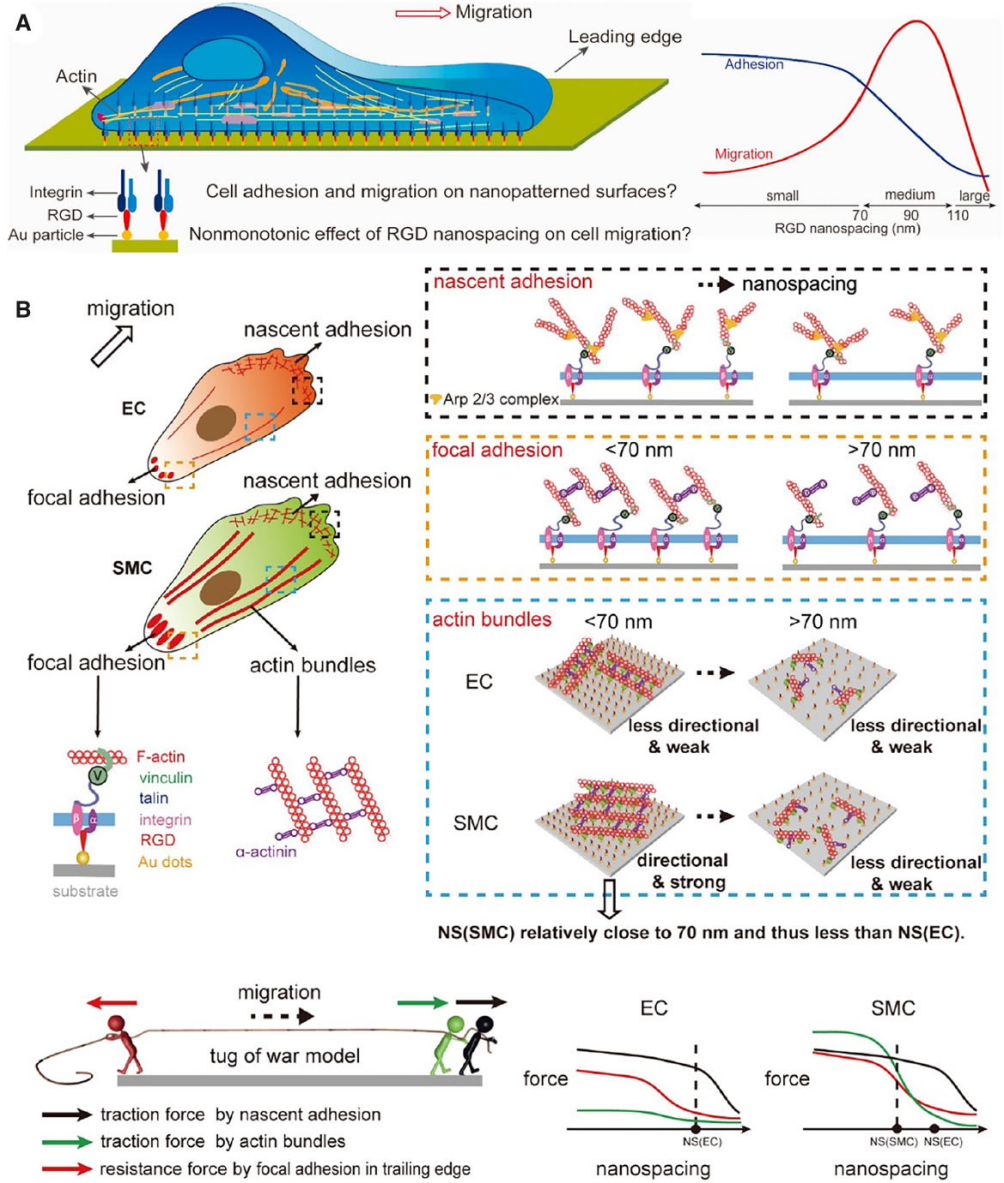
The effects of nanopattern on cell adhesion and migration. (**A**) The effects of RGD nanospacing on cell migration and its relation to cell adhesion. Reproduced from Ref. [411] with permission of Elsevier, © 2020. (**B**) The migration of different type cells on biomaterials adjusted by RGD nanospacing. Reproduced from Ref. [412] with permission of American Chemical Society, © 2021.

Appropriate cell migration can ensure the integration of a medical implant with its surrounding tissue while inadequate or excessive migration leads to adverse reactions. Rapid endothelialization reduces the occurrence rate of thrombosis and restenosis, which is particularly of importance for the cardiovascular stents [406]. Endothelialization is closely related to cell adhesion and migration. Ding *et al*. [407–410] prepared a series of nanopatterns with varied RGD nanospacings. They further examined the effects of RGD nanospacing on the adhesion and migration of human umbilical vein endothelial cells (HUVECs) [411]. Interestingly, while adhesion of HUVECs on RGD nanopatterns changed with RGD nanospacings monotonically, the maximum migration velocity was observed in the nanopattern of RGD nanospacing around 90 nm (Fig. 13A). This finding indicates the importance of surface modification on the nanoscale—an appropriate surface modification can enhance the expected effect for a regenerative biomaterial, and an inappropriate nanoscale modification may lead to the opposite effect.

A new question arises, whether the cell type influences the regulation of RGD nanospacing on cell adhesion and migration. Ding *et al*. [412] employed well-designed RGD nanopatterns to investigate the effect of the surface modification on cell migration using ECs and smooth muscle cells (SMCs) as demonstrated cell types (Fig. 13B). Their results indicated the cell-type-dependent migration peak of RGD nanospacing, which was interpreted from the different resistances to detach a focal adhesion plaque at the trailing edge as well as the different abilities to form a nascent adhesion at the leading edge of a polarized cell on a material. Ding’s group further revealed the cell selective migration in a gradient nanopattern of RGD ligands prepared by them [413], and they found that a cell, particularly a human SMC could sense the difference of RGD nanospacing on the surface with even one nanometer between leading and trailing edges along the cell polarization dimension.

In addition to the regulation of biomaterial surfaces, some other factors of biomaterials can also effectively regulate cell migration and are of great significance, such as pore architecture [414, 415], geometry [416], stiffness [417–420] and mechanical stimulus [421, 422]. For example, Zou *et al*. [423] found that human dental pulp cells adhered better on the scaffolds with 400–600 μm pore sizes than on the scaffolds with 700 μm pore sizes. The cells would directly pass through the large pore of the scaffolds, resulting in a decrease of cell adhesion.

Cell differentiation is crucial for tissue regeneration. The microenvironment around stem cells is able to precisely control the developmental processes of organism via particular cues [424, 425]. Therefore, understanding which cues can regulate the differentiation of stem cell provides key insights into the development of tissue regeneration [426, 427]. Biomaterials combined with advanced techniques can mimic the *in vivo* microenvironment to drive the cell differentiation to a desirable direction or adjust the function of a cell [428–430]. One has designed model biomaterials to study the mechanism how each cue affects stem cells [431, 432]. For example, mechanical forces can influence cell behavior via regulating their intracellular signal pathways [433]. Yao *et al*. [434] developed a platform to investigate the influence of fluid shear stress plus ultrasound stimulation on the behavior of bone marrow MSCs. The results showed that cell differentiation was responsive to the magnitudes of fluid shear stress and ultrasound stimulations and thus significantly enhanced by proper combination strategies.

Wei *et al*. [435] investigated the effects of topographical cues on cell polarization via fabricating a material with orthogonal microgrooves and nanogrooves that mimic the effects of space constraint and adhesion induction. Specifically, space constraint in a microstructure is introduced to limit cell growth via guiding cell elongation in a direction. Adhesion induction is to guide adhesome growth via using nanopatterns at the interface of materials. They found that the osteogenic differentiation of stem cells can be promoted by the enhanced intracellular force caused by adhesion induction.

### Biomaterials that modulate immune cells

Foreign body responses are critical for implantable biomaterials. The response process contains protein adsorption, recruitment of immune cells and formation of fibrous capsule etc. [436, 437]. This response used to be recognized always as a side effect of a biomaterial [438]. Therefore, early design of a biomaterial focused on minimizing the immune response in order to decrease body rejection [439, 440]. Now, a new insight emerges toward ‘immune-interactive’ biomaterials in the field of regenerative medicine [441].

Immune cells are divided into two types—pro-inflammatory cells and anti-inflammatory cells [442]. The balance of the different types of immune cells determines the outcome of the tissue regeneration [443]. ‘Immune-interactive’ biomaterials can modulate such a balance and thereby direct the suitable immune response for tissue regeneration [444, 445]. Some biomaterials that are capable of mediating macrophage polarization are particularly attractive for tissue regeneration [446]. Macrophages can polarize to pro-inflammatory M1 phenotype or anti-inflammatory M2 phenotype. The two phenotypes of macrophages work differently in regeneration of various tissues [447]. M2 macrophages enhance bone repairing via cytokine production whereas M1 macrophages work conversely [448]. For tumor treatment, M1 macrophages are important for antigen presentation, which helps the immune cells to attack tumor cells. Conversely, M2 macrophages correlate with tumor growth and metastasis. Therefore, the ‘immune-interactive’ biomaterials are designed to induce the polarization of tumor-associated macrophages to the M1 phenotype. For example, Gu *et al*. [449] developed a postoperative sprayed gel loaded with calcium carbonate nanoparticles and anti-CD47 antibody to polarize the macrophages to M1 phenotype by scavenging H^+^ in the surgical site.

**Figure 14.**
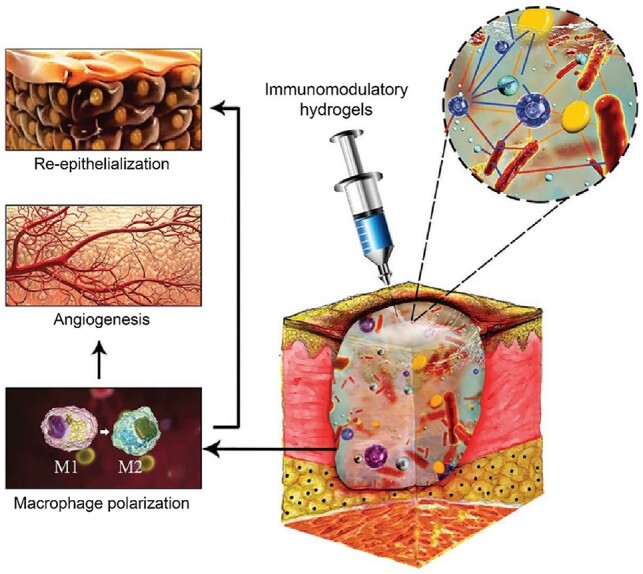
Immunomodulatory hydrogel. Reproduced from Ref. [450] with permission of Wiley-VCH, © 2021.

For the wound healing, M2 macrophages should replace the M1 macrophages (work in the initial stage) to promote tissue repair by releasing anti-inflammatory cytokines, which has inspired the development of an immunomodulatory hydrogel (Fig. 14) [450]. The ‘immune-interactive’ biomaterials take advantage of many strategies, including adjusting the pore size, stiffness and surface topography of biomaterials, to help the regeneration via regulating macrophages polarization [451, 452]. Besides, the bioactive factors or ions released from biomaterials can also tune the ratio of M2/M1 macrophages [453, 454].

## Summary and perspective

Regenerative biomaterials have been developed significantly in recent years [155, 220, 455–457]. The trend of biomaterial development has evolved from bio-inert materials to bioactive materials [10, 458–461]. The major material types of regenerative biomaterials including metals, ceramics, polymers and bio-derived materials have been explored extensively, and each type of biomaterials has the advantages and disadvantages. Metallic materials usually have an excellent mechanical property and are very useful in the cases strongly demanding reliable mechanical support. While the dissolved bioactive ions may endow more functions to biomaterials, the potential side effects in directing cellular response should be considered in medical applications. The bioactive inorganic materials including bioceramics and bioglass have succeeded in orthopedic tissue regeneration owing to their osteoinductive activity. The brittleness is the main weakness of most of inorganic biomaterials, which may be improved to some extents. Hydrogels can, as an outstanding soft material, perfectly match the biomechanics of soft tissues, and the high-water content and 3D networks endow the capacity of drug delivery [462]. Other polymer materials, especially biodegradable polymers, show great potential for application in human body [463, 464]. One of the advantages of polymer materials is that the performance can be precisely mediated by molecular engineering [465]. For bio-derived materials, the immunogenicity is the biggest obstacle for clinical translation, while the structure of the ECM of bio-derived materials makes them quite unique in some cases. Fundamental studies involving cell-biomaterial interactions can guide biomaterial design [58, 466–468]. And the advanced technologies, such as 3D bio-printing, have paved the way for fabricating innovative biomaterials.

**Figure 15.**
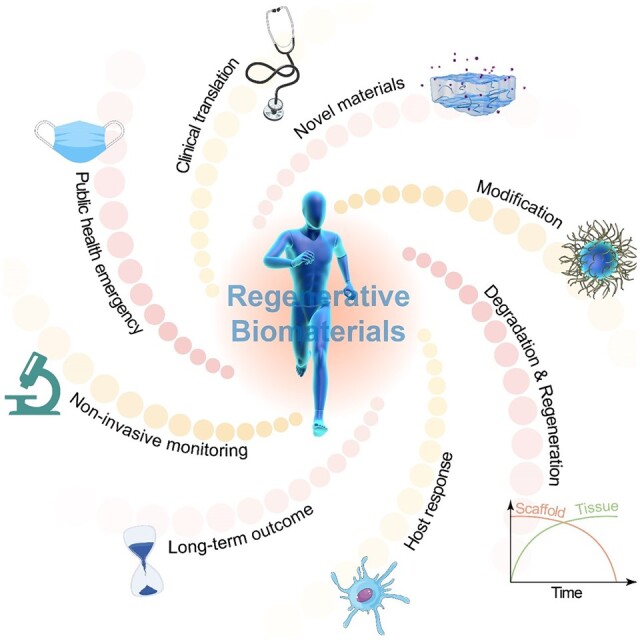
Perspective key issues regarding the development of regenerative biomaterials.

With the state-of-the-art advances of materials and the in-depth exploration of cell-biomaterial interactions, regenerative biomaterials have achieved exciting outcomes. However, there are still challenges in the field of regenerative biomaterials. In our opinion, there are some critical points that should be carefully considered in the future, as schematically presented in Fig. 15.

1. Creation of new materials is the source of innovation in regenerative biomaterials. Molecular engineering allows the precise design of biomaterials. For example, some novel polymeric materials can respond to specific external stimuli via chemical linking of stimulus-generating moieties. The collaboration of chemists and material scientists will be efficient to promote regenerative biomaterials.
2. Modification of existing materials is an effective strategy for improving the material performances. Different types of materials have their own advantages, which can sometimes be unified into one device to improve the entire performance. Advanced techniques of biofabrication of regenerative scaffolds should also be paid attention to [469]. Moreover, leveraging advanced technology to modify the existing materials can endow them fresh functions for extensive applications [470].
3. Biomaterial degradation and tissue regeneration are required to be harmonious with each other. Utility of biodegradable raw materials have been the mainstream in the development of many new medical devices and DDSs. Then it is important to regulate the biodegradable profiles [471, 472]. An ideal regenerative biomaterial could be designed to have the degradation profile match the regeneration process of the targeted tissue in a specific microenvironment. Besides, the *in vitro* and *in vivo* degradation rates and profiles of a regenerative implant might be different [473, 474], and in some cases, the results differ with characterization methodologies *in vitro* [475, 476]. The degraded product of an ideal regenerative biomaterial should be non-toxic. Even the degradation rate has recently been found to afford a ‘dynamic cue’ to regulate stem cells beyond varied matrix stiffness [477]. All of these issues should be taken into consideration.
4. Host responses can significantly influence the clinical outcomes of the biomaterial. Cell-material interactions are particularly important after the concept of ‘materiobiology’ has been put forward [478]. The *in vivo* fate is crucial for a regenerative biomaterial and can be significantly influenced by the host responses [479]. The design of a regenerative biomaterial should consider the therapeutic outcomes and the *in vivo* change of the material itself. Researchers have gradually realized the importance of immunomodulatory effects of regenerative biomaterials [480]. Thereby more strategies are expected to be developed to induce a favorable immune environment after implantation of a regenerative biomaterial.
5. The long-term outcomes of regenerative biomaterials should be paid more attention to. Since some novel biomaterials just appeared, the long-term outcomes are still inconclusive. More preclinical studies and biosafety evaluation are necessity for R & D of a biomaterial. What is more challenging and expensive is the *in vivo* examination of the long-term outcome. The translation gap between animals and human seemed to be underestimated previously, and the efficacy of biomaterials between different species might be significantly different in many cases. The authors strongly call for the pertinent R & D centers, and those established large research groups are encouraged to spend more time and money to solve these tough problems and undertake the expensive mission.
6. The noninvasive approaches for monitoring *in vivo* dynamic evolution of implanted materials and tissue regeneration are required to be developed. The final goal of biomaterial development is clinical translation. While some methods have been developed to observe and evaluate the degradation process *in vitro* [481], the final evaluation of a biomaterial still needs to be based on the *in vivo* and even clinical data. Without suitable and precise noninvasive monitor approaches, the efficacy of biomaterials in human could not be acquired. These facts remind us of the importance of noninvasive evaluation systems, such as MRI [482], fluorescence imaging [483], ultrasound imaging [484, 485], computed tomography [486], positron emission tomography [487] and X-ray imaging [488]. More noninvasive imaging technologies need to be developed to access a biomaterial for clinical translation.
7. Public health emergencies call for more R & D of biomaterials. The situation of the COVID-19 pandemic reminds us that modern human being needs high-value flexible solutions to urgent clinical requirement. In this regard, people and government need a solid foundation of biomaterials to offer the tools for the quick and effective responses to global public health emergency in terms of prevention, diagnosis and treatment of various diseases. A biomaterial particularly worthy of attention is lipid nanoparticle, which has been used in the mRNA vaccine of COVID-19 and recently been well reviewed by Langer and Dong groups [375]. Nevertheless, there is a big space to develop the nanotechnology to enhance the vaccine efficacy. Besides COVID-19 [489], HIV [490] and Ebola virus [491] still exist seriously in the world. More recently, monkeypox, a novel infectious disease caused by zoonotic monkeypox virus, has emerged community transmission outside of the African continent in 2022, which has raised concern about large-scale global spread [492, 493]. It is worthy of warning that two dangerous epidemic diseases, cholera [494] and pestis [495], which have ever seriously destroyed human being [496], comeback occasionally [497, 498]. Human being should prepare sufficient biomaterial techniques to deal with the potential new pandemic emergency to protect human being.
8. Clinical translation needs to be pushed forward in a full-chain way. In the following years, one of critical directions of the development of regenerative biomaterials is the clinical translation. Without a real application for the benefit of mankind, the biomaterials could only be a work of art albeit exquisite design. For example, nanotechnology has held significant promise in the field of regenerative biomaterials, yet only a few nanomedicines have achieved clinical translation partly due to the limited efficacy in human studies compared with the successful outcomes in animal models [331]. Similarly, there are much less clinical translations of regenerative biomaterials compared to numerous publications. Hence, the bridge between fundamental studies and clinical translations of regenerative biomaterials needs to be built and reinforced. Quite a number of researchers are encouraged to be dedicated in the translational research. With the emerge of novel materials and technologies, the corresponding standard and qualification must keep up to access and control the manufactured biomaterials. A successful translation of a biomaterial involves a full industrial chain from clinical trial to transportation, shelf-life and packaging. Actually, even a tiny problem may lead to a failure of an entire clinical translation. Only the researchers understand the clinical demand to some extents, can they transform research innovations into new products or treatments. The regenerative biomaterial fields should focus more time and resource toward translational research. We are confident that many of the obstacles in the path of the translation can be circumvented upon the development of biomaterials for medicine and pharmacy. Last but not least, capital and government supports are important to promote the translation of a regenerative biomaterial.

In summary, regenerative biomaterials have been an interdisciplinary frontier. More new insights will be shed onto this brilliant field. Artificial intelligence (AI) and brain science will revolutionize human life and global economics [499–501] and can be combined with regenerative biomaterials for advanced medical devices and DDSs. We believe that regenerative biomaterials can assist to reveal some sciences in the fields of AI and brain science, and biomaterial scientists will employ the new achievements of these fields to further develop regenerative biomaterials in the future.
